# Regulation of c-SMAC formation and AKT-mTOR signaling by the TSG101-IFT20 axis in CD4^+^ T cells

**DOI:** 10.1038/s41423-023-01008-x

**Published:** 2023-04-07

**Authors:** Jiung Jeong, In Kang, Yumin Kim, Keun Bon Ku, Jang Hyun Park, Hyun-Jin Kim, Chae Won Kim, Jeongwoo La, Hi Eun Jung, Hyeon Cheol Kim, Young Joon Choi, Jaeho Kim, Joon Kim, Heung Kyu Lee

**Affiliations:** 1 Graduate School of Medical Science and Engineering, Korea Advanced Institute of Science and Technology (KAIST), Daejeon, 34141 Republic of Korea; 2 Department of Internal Medicine, Seoul National University Hospital, Seoul, 03080 Republic of Korea; 3 Department of Biological Sciences, KAIST, Daejeon, 34141 Republic of Korea; 4 Department of Convergent Research of Emerging Virus Infection, Korea Research Institute of Chemical Technology, Daejeon, 34114 Republic of Korea

**Keywords:** CD4^+^ T cell, Intraflagellar transport 20, Tumor susceptibility gene 101, AKT-mTOR signaling, Asthma, Supramolecular activation cluster, T-helper 2 cells, Lymphocyte activation

## Abstract

CD4^+^ T cells play major roles in the adaptive immune system, which requires antigen recognition, costimulation, and cytokines for its elaborate orchestration. Recent studies have provided new insight into the importance of the supramolecular activation cluster (SMAC), which comprises concentric circles and is involved in the amplification of CD4^+^ T cell activation. However, the underlying mechanism of SMAC formation remains poorly understood. Here, we performed single-cell RNA sequencing of CD4^+^ T cells left unstimulated and stimulated with anti-CD3 and anti-CD28 antibodies to identify novel proteins involved in their regulation. We found that intraflagellar transport 20 (IFT20), previously known as cilia-forming protein, was upregulated in antibody-stimulated CD4^+^ T cells compared to unstimulated CD4^+^ T cells. We also found that IFT20 interacted with tumor susceptibility gene 101 (TSG101), a protein that endocytoses ubiquitinated T-cell receptors. The interaction between IFT20 and TSG101 promoted SMAC formation, which led to amplification of AKT-mTOR signaling. However, IFT20-deficient CD4^+^ T cells showed SMAC malformation, resulting in reduced CD4^+^ T cell proliferation, aerobic glycolysis, and cellular respiration. Finally, mice with T-cell-specific IFT20 deficiency exhibited reduced allergen-induced airway inflammation. Thus, our data suggest that the IFT20-TSG101 axis regulates AKT-mTOR signaling via SMAC formation.

## Introduction

CD4^+^ T cells are the major players of the immune system. These cells recognize peptides bound to the major histocompatibility complex (MHC) class II on antigen-presenting cells (APCs) and orchestrate the activities of downstream target cells (e.g., cytotoxic T cells, eosinophils, and neutrophils), stimulating them to release various cytokines [1]. Malfunction of CD4^+^ T cells is associated with many diseases. For example, hyperactivation of CD4^+^ T cells can result in asthma and autoimmune disease, whereas hypoactivation of these cells can lead to immunodeficiency syndrome [2–4]. Although research suggests a role for CD4^+^ T cells in these diseases and their progression, a therapeutic agent using CD4^+^ T cells has yet to be successfully developed.

Intraflagellar transport (IFT) proteins act as transporters to move nonmembrane-bound particles [5]. IFTs play a central role in the assembly and maintenance of cilia, as several IFT proteins are combined to form two subcomplexes, IFT-A and IFT-B [5]. Intraflagellar transport 20 (IFT20), which binds IFT54 and IFT57, is a central protein that connects IFT-A and IFT-B [6]. IFT20 is localized mainly in the Golgi apparatus and moves to the cilia during ciliogenesis [7]. In osteoblasts, IFT20 is involved in collagen deposition by trafficking from the endoplasmic reticulum to the Golgi apparatus [8]. IFT20 deficiency leads to significant impairment in cilia formation and has been associated with various cilia-related diseases, such as retinal degeneration, polycystic kidney disease, and azoospermia [9–11]. As research on IFT20 has been limited to cilia formation and cilia-related disease, the role of IFT20 in nonciliated cells, such as immune cells, is not well understood.

Here, to identify novel proteins involved in the regulation of CD4^+^ T cells, we performed single-cell RNA sequencing (scRNA-seq) on CD4^+^ T cells left unstimulated and stimulated with anti-CD3 and anti-CD28 antibodies. Surprisingly, IFT20 was upregulated in stimulated CD4^+^ T cells compared to unstimulated CD4^+^ T cells. We found a direct protein‒protein interaction between IFT20 and tumor susceptibility gene 101 (TSG101), an endocytosis-related protein. Interaction between IFT20 and TSG101 amplified phosphorylation of AKT-mTOR. In addition, IFT20 was found to promote aerobic glycolysis and cellular respiration by regulating glucose transporter 1 (GLUT1) expression. Conversely, IFT20 deficiency in CD4^+^ T cells led to downregulation of AKT-mTOR signaling, resulting in reduced CD4^+^ T cell proliferation, aerobic glycolysis, and cellular respiration. Using two murine models of allergen-induced allergen, we demonstrated that CD4^+^ T cell-specific conditional IFT20-knockout (KO) mice exhibited attenuated allergic airway inflammation.

## Materials and methods

### Mouse lines

To generate mice with a CD4^+^ T cell-specific conditional IFT20-knockout (KO) phenotype, we mated IFT20^f/f^ mice (B6.129S7(129S4)-Ift20^tm1.1Gjp^/J) that were obtained from the Jackson Laboratory with CD4-Cre mice (B6.Cg-Tg(Cd4-cre)1Cwi/BfluJ). The CD4^+^ T cell-specific conditional TSG101-KO phenotype was generated by mating TSG101^f/f^ mice (TSG101^tmKuw^, MGI:2447442) obtained from the Mutant Mouse Resource & Research Center with CD4-Cre mice. We used 5- to 10-week-old male IFT20^f/f^, IFT20^f/f^ CD4-Cre, TSG101^f/f^, and TSG101^f/f^ CD4-Cre mice in all experiments. The mice were bred and maintained in a specific pathogen-free facility of the KAIST Laboratory Animal Resource Center. All procedures were performed according to the guidelines and protocols (KA2018-47 and KA2020-125) for rodent experimentation provided by the Institutional Animal Care and Use Committee at our institution.

### Papain-induced airway inflammation model

Mice were administered 25 μg papain (Merck, Darmstadt, Germany) intranasally for three consecutive days to sensitize them to the allergen. On Day 14 after the first administration of papain, mice were challenged with the same concentration of papain administered via intranasal injection. Bronchoalveolar lavage (BAL) fluid and cells were harvested from lung tissue on Day 15, and IL-5 and IL-13 from BAL fluid were quantified later by enzyme-linked immunosorbent assay (ELISA). Some lung tissue samples were prepared as slides and subjected to hematoxylin and eosin (H&E) staining. Cells obtained from the remaining lung tissue were analyzed with flow cytometry as described below.

### Ovalbumin (OVA)-induced airway inflammation model

Mice were administered 100 μg of OVA (Sigma‒Aldrich, St. Louis, MO, USA) and 4 mg of alum (Thermo Fisher Scientific, Waltham, MA, USA) via intraperitoneal injection on Day 0. Then, mice were intranasally challenged with 50 μg of OVA on Days 7–9 after the first intraperitoneal injection of OVA and alum, and lung tissue was harvested on Day 10. Some lung tissue samples were prepared as slides and subjected to H&E staining. Cells obtained from the remaining lung tissue were analyzed by flow cytometry as described below.

### Measurement of airway resistance

Mice were anesthetized by intraperitoneal administration of ketamine (Yuhan Corporation, Seoul, Korea) and xylazine (Bayer, Leverkusen, Germany). After tracheostomy using a 20-gauge cannula, mice were connected to a flexiVent (SCIREQ, Montréal, Canada) with a respiratory rate of 150 breaths/min and tidal volume of 10 mL/kg against a positive end-expiratory pressure of 3 cm H_2_O. Then, airway resistance was measured by the snapshot perturbation maneuver while increasing the concentration of methacholine (Sigma‒Aldrich, St. Louis, MO, USA).

### Flow cytometry

Single cells were collected from mouse spleen and lung tissues and then washed with cold wash buffer composed of Dulbecco’s phosphate-buffered saline (DPBS) with 1% bovine calf serum and 1% penicillin‒streptomycin. The cells were incubated for 30 min on ice with the appropriate antibodies. Anti-CD16/32 antibody was added to the fluorophore-conjugated antibody mixture to block Fc receptors. Single cells were stained with fluorophore-conjugated antibodies and stained as described in Supplementary Table 1. Flow cytometry analyses for all samples were performed using FACSCalibur or LSRFortessa cell analyzers (Becton Dickinson, Franklin Lakes, NJ, USA), and data were analyzed with FlowJo software (Becton Dickinson, Franklin Lakes, NJ, USA).

### In vitro CD4^+^ T-cell activation

CD4^+^ T cells for T-cell activation were extracted from the spleens of experimental and control mice. We used a Magnisort^TM^ Mouse CD4 Naïve T cell Enrichment Kit (Invitrogen, Waltham, MA, USA) to isolate purified naïve T cells. The purity of the enriched CD3^+^CD4^+^CD44^lo^ T cells was 90–95%. Naïve CD4^+^ T cells were stimulated with 1 μg/mL anti-CD3 (Invitrogen, Waltham, MA, USA) and 1 μg/mL anti-CD28 antibodies (BioLegend, San Diego, CA, USA). The phosphorylation of AKT1, mTOR and RPS6KB1 was assessed by flow cytometry. For TSG101-deficient CD4^+^ T cell analysis, whole CD4^+^ T cells from the spleens of experimental or control mice were enriched using a Magnisort™ Mouse CD4 T cell Enrichment Kit (Invitrogen, Waltham, MA, USA) and stimulated with 1 μg/mL anti-CD3 and 1 μg/mL anti-CD28 antibodies for 8 h. Activated CD4^+^ T cells were harvested and analyzed by flow cytometry. The antibodies used for flow cytometry are listed in Supplementary Table 1.

### Western blotting and coimmunoprecipitation (Co-IP)

CD4^+^ T cells or non-CD4^+^ T cells for identification of IFT20 were extracted from the spleens of experimental and control mice. We used the Magnisort^TM^ Mouse CD4 T-cell Enrichment Kit (Invitrogen, Waltham, MA, USA) to isolate purified CD4^+^ T cells or non-CD4^+^ T cells. The purity of the enriched CD4^+^ T cells was determined to be 90–95%. CD4^+^ T cells or non-CD4^+^ T cells were lysed with RIPA buffer with Xpert Protease Inhibitor Cocktail Solution (GenDEPOT, Katy, TA, USA). Next, 500 μg samples of lysates of enriched CD4^+^ T cells from experimental and control mice were loaded into the wells of Mini-PROTEAN TGX Stain-Free Precast Gels (Bio-Rad, Hercules, CA, USA).

HEK293T cells for Co-IP were transfected with strep-tag II tagged IFT20 plasmid (Korea Human Gene Bank, Clone ID NU007764) and TSG101 plasmid (Korea Human Gene Bank, Clone ID mMU001071) using Lipofectamine™ 3000 Transfection Reagent (Invitrogen, Waltham, MA, USA) for 48 h. Transfected HEK293T cells that overexpressed IFT20 and TSG101 were lysed with 1% Triton™ X-100 (Sigma‒Aldrich, St. Louis, MO, USA). The strep-tag II pooled protein samples were obtained from 1000 μg of lysates of HEK293T cells using SureBeads™ Protein A Magnetic Beads (Bio-Rad, Hercules, CA, USA) according to the manufacturer’s protocol.

Jurkat cells (Korea Cell Line Bank, Clone ID E6-1) used for Co-IP were stimulated with 1 μg/mL anti-CD3 (Invitrogen, Waltham, MA, USA) and 1 μg/mL anti-CD28 antibodies (Becton Dickinson, Franklin Lakes, NJ, USA) for 24 h. Then, unstimulated and stimulated Jurkat T cells were lysed with 1% Triton™ X-100 (Sigma‒Aldrich, St. Louis, MO, USA). IFT20 pooled protein samples were obtained from 500 μg of lysates of Jurkat cells using SureBeads™ Protein A Magnetic Beads (Bio-Rad, Hercules, CA, USA) according to the manufacturer’s protocol.

The pooled protein samples were loaded into the wells of Mini-PROTEAN TGX Stain-Free Precast Gels. After electrophoresis at 60 V for 1.5 h, the proteins in each well were transferred to a Trans-blot Turbo Mini 0.2 μm PVDF membrane using Trans-Blot Turbo Transfer (Bio-Rad, Hercules, CA, USA). The transferred PVDF membrane was incubated with a 1:500 dilution of IFT20 antibody, a 1:500 dilution of TSG101 antibody (Abcam, Cambridge, UK) and a 1:5000 dilution of GAPDH antibody (Cell Signaling, Danvers, MA, USA) overnight at 4 °C. The next day, the PVDF membrane was stained with secondary StarBright Blue 700 goat anti-rabbit IgG and StarBright Blue 700 goat anti-mouse IgG (Bio-Rad, Hercules, CA, USA) at a 1:5000 dilution for 1 h at room temperature. The levels of IFT20, TSG101 and GAPDH were measured with a ChemiDoc^TM^ MP Imaging System (Bio-Rad, Hercules, CA, USA). The antibodies used for western blotting and co-IP are listed in Supplementary Table 2.

### ELISA

BAL fluids from the papain-induced airway inflammation model and supernatants acquired after in vitro CD4^+^ T cell activation and differentiation were stored at −80 °C. The quantities of cytokines were measured using the antibodies listed in Supplementary Table 3. We used an iMark microplate absorbance reader and MPM 6 software (Bio-Rad, Hercules, CA, USA) to determine the absorbance of BAL fluids and supernatants and to calculate cytokine levels, respectively.

### Quantitative real-time polymerase chain reaction (qRT‒PCR)

Total RNA was isolated from CD4^+^ T cells using an RNeasy Plus Mini Kit (Qiagen, Hilden, Germany) according to the manufacturer’s protocol. ReverTra Ace qPCR RT Master Mix (TOYOBO, Osaka, Japan) was used for cDNA synthesis. We measured the Ct value of each sample using a CFX96 Real-Time PCR system (Bio-Rad, Hercules, CA, USA) with SYBR Green-based quantification (TOYOBO, Osaka, Japan). The amount of RNA was normalized relative to *hypoxanthine phosphoribosyltransferase (Hprt)* expression, and the relative fold difference for unstimulated wild-type (WT) CD4^+^ T cells is indicated. The primers used for qPCR analysis were as follows: *Ift20* (forward: 5ʹ- AGA AGC AGA GAA CGA GAA GAT G-3ʹ; reverse: 5ʹ- CAC AAA GCT TCA TAT TCA ACC CG-3ʹ) [12]; *Tsg101* (forward: 5ʹ- TCT AAC CGT CCG TCA AAC TGT-3ʹ; reverse: 5ʹ- TTG TAC CAG TGA GGT TCA CCA-3ʹ) [13]; *Hprt* (forward: 5ʹ- GTT GGA TAC AGG CCA GAC TTT GTT G-3ʹ; reverse: 5ʹ- GAG GGT AGG CTG GCC TAT TG GCT-3ʹ) [14].

### Glycolysis stress test

Naïve CD4^+^ T cells were either left unstimulated or stimulated with 1 μg/mL anti-CD3 and 1 μg/mL anti-CD28 antibodies for 48 h. Approximately 400,000 unstimulated CD4^+^ T cells and stimulated CD4^+^ T cells were plated in XF Medium, which was composed of nonbuffered Dulbecco’s modified Eagle medium (DMEM) and 2 mM L-glutamine (Thermo Fisher Scientific, Waltham, MA, USA). Using an XFe96 Extracellular Flux Analyzer (Agilent Technologies, Santa Clara, CA, USA), the extracellular acidification rate (ECAR) was measured by reaction with 10 mM glucose (WELGENE, Gyeongsan, Korea), 2.5 µM oligomycin (Merck, Darmstadt, Germany), and 50 µM 2-DG (Sigma‒Aldrich, St. Louis, MO, USA). ECAR data were analyzed with Wave software (Agilent Technologies, Santa Clara, CA, USA).

### Mitochondrial stress test

Naïve CD4^+^ T cells were either left unstimulated or stimulated with 1 μg/mL anti-CD3 and 1 μg/mL anti-CD28 antibodies for 48 h. Unstimulated CD4^+^ T cells and stimulated CD4^+^ T cells were plated in XF Medium, which was composed of nonbuffered Dulbecco’s modified Eagle medium (DMEM) with 2 mM L-glutamine, 25 mM glucose, and 1 mM pyruvate (Sigma‒Aldrich, St. Louis, MO, USA). Using an XFe96 Extracellular Flux Analyzer (Agilent Technologies, Santa Clara, CA, USA), the oxygen consumption rate (OCR) were measured by reaction with 1 µM oligomycin, 1.5 µM carbonyl cyanide 4-(trifluoromethoxy) phenylhydrazone (Sigma‒Aldrich, St. Louis, MO, USA), 0.1 µM rotenone (Sigma‒Aldrich, St. Louis, MO, USA), and 1 µM antimycin A (Sigma‒Aldrich, St. Louis, MO, USA). OCR data were analyzed with Wave software.

### Single-cell RNA sequencing (scRNA-seq)

Approximately 200,000 unstimulated 7AAD^-^CD3^+^CD4^+^ T cells and CD4^+^ T cells stimulated with anti-CD3 and anti-CD28 antibodies were sort-purified using a FACSAria^TM^ II flow cytometer (Becton Dickinson, Franklin Lakes, NJ, USA). Of these, 10,000 cells that passed quality control were used to produce a single-cell RNA library generated using the Chromium Single Cell 3ʹ GEX Library & Gel Bead Kit v3 (10x Genomics, Pleasanton, CA, USA) according to the manufacturer’s instructions. To generate FASTQ files, sequence data were demultiplexed using cellranger mkfastq in Cell Ranger v.3.0.2 (10x Genomics, Pleasanton, CA, USA). We then used cellranger count to take FASTQ files from cellranger mkfastq and perform sequence alignment, filtering, barcode counting, and unique molecular identifier counting. Sequencing data were analyzed with R software using the Seurat algorithm. To exclude low-quality cells, we selected cells expressing >200 genes, of which <15% were mitochondrial genes. Data corresponding to each cell were normalized, and approximately 1500–3000 genes from each dataset were identified to align cells originating from different samples.

### Yeast two-hybrid (Y2H) analysis

Y2H screening of IFT20 and TSG101 bait plasmids was performed in the *Saccharomyces cerevisiae* AH109 strain, which contained two reporters (*HIS3* and *ADE2*) controlled by different *GAL* promoters. Yeast transformants containing the IFT20 bait plasmid (Korea Human Gene Bank, Clone ID NU007764), the TSG101 bait plasmid (Korea Human Gene Bank, Clone ID mMU001071), and the spleen cDNA activation domain (AD) library from mice and humans were spread on selective media. Yeast colonies growing on selective media were chosen based on reporter gene activity. Protein‒protein interactions were tested using two independent reporters with different types of GAL4-binding sites. To confirm the interaction, the prey portion of the plasmid DNA from 60 H^+^A^+^ candidates was amplified by PCR or by *Escherichia coli* transformation. Amplified candidate prey portions were then reintroduced into yeast using a bait plasmid, a positive control plasmid, or a negative control plasmid.

### Confocal microscopy

Stimulated CD4^+^ T cells were washed twice with 1 mL of DPBS. After washing, 1 × 10^5^ stimulated CD4^+^ T cells were attached to glass slides for 3 min at 2,000 rpm using Cytospin^TM^ 4 (Thermo Fisher Scientific). Slides containing CD4^+^ T cells were fixed with 4% formaldehyde for 30 min at room temperature and stained overnight at 4 °C with a primary antibody mixture. The following day, the slides were stained with a secondary antibody mixture for 30 min at room temperature. Immunofluorescence images were acquired using the Airyscan mode of a Zeiss LSM 800 confocal microscope (Carl Zeiss, Oberkochen, Germany). ZEN 3.2 software (Carl Zeiss, Oberkochen, Germany) was used to measure the signal intensity of each cell. The antibodies used for immunofluorescence staining are listed in Supplementary Table 4.

### Transmission electron microscopy (TEM)

Spleens were extracted from WT mice. Following single-cell isolation, splenocytes were fixed with 2.5% glutaraldehyde in 0.1 M DPBS for 2 h at 4 °C and then postfixed for 2 h in 2% osmium tetroxide. In the process of gradually increasing the concentration of ethanol and propylene oxide, the fixed samples were dehydrated and finally embedded in EMbed-812 resin (Electron Microscopy Sciences, Hatfield, PA, USA). Polymerization was performed at 60 °C for 24 h, and an ultramicrotome (Leica, Wetzlar, Germany) was used to prepare ultrathin (100 nm) sections.

For immunogold labeling, prepared sections were reacted with etching solutions for 1 h and then blocked with Tris-buffered saline containing 4% goat serum, 1% bovine serum albumin, and 0.1% Tween-20. The sections were incubated with a 1:100 dilution of IFT20 antibody overnight at 4 °C. The next day, the sections were stained with a gold-conjugated goat anti-rabbit IgG secondary antibody (AURION, Wageningen, Netherlands) at a 1:100 dilution for 2 h at room temperature. Finally, the sections were sequentially dyed with 2.5% uranyl acetate for 7 min and lead citrate for 3 min. Images were captured with a Tecnai G2 spirit TWIN TEM (FEI Company, Hillsboro, OR, USA) at an acceleration voltage of 120 kV. The antibodies used for immunogold labeling are listed in Supplementary Table 4.

### Statistical analysis

The data are expressed as the mean ± standard error of the mean (SEM). Unpaired or paired Student’s *t* tests were used to analyze differences between groups, with *P* < 0.05 considered to indicate statistical significance. SEM and *P* values were calculated using Prism v.8.00 (GraphPad, San Diego, CA, USA).

## Results

### IFT20 is important for CD4^+^ T cell activation

To identify genes that are essential for CD4^+^ T cell activation, we performed scRNA-seq on unstimulated and stimulated CD4^+^ T cells that were isolated from the spleens of wild-type (WT) mice. CD4^+^ T cell activation via treatment with anti-CD3 and anti-CD28 antibodies for 24 h was confirmed by measuring the expression of effector T cell markers, namely, CD44 and CD69 upregulation (Supplementary Fig. 1A, B). Approximately 200,000 7-amino actinomycin D (7AAD)^-^CD3^+^CD4^+^ cells were sort-purified from unstimulated CD4^+^ T cells (none) or stimulated with anti-CD3 and anti-CD28 antibodies (CD3/CD28). Among these, 10,000 cells that passed quality control were used to produce a single-cell RNA library. We identified 1665 and 2981 differentially expressed genes (DEGs) per cell in the “none” and “CD3/CD28” populations, respectively, with more than 15,000 different genes observed per sample.

**Fig. 1:**
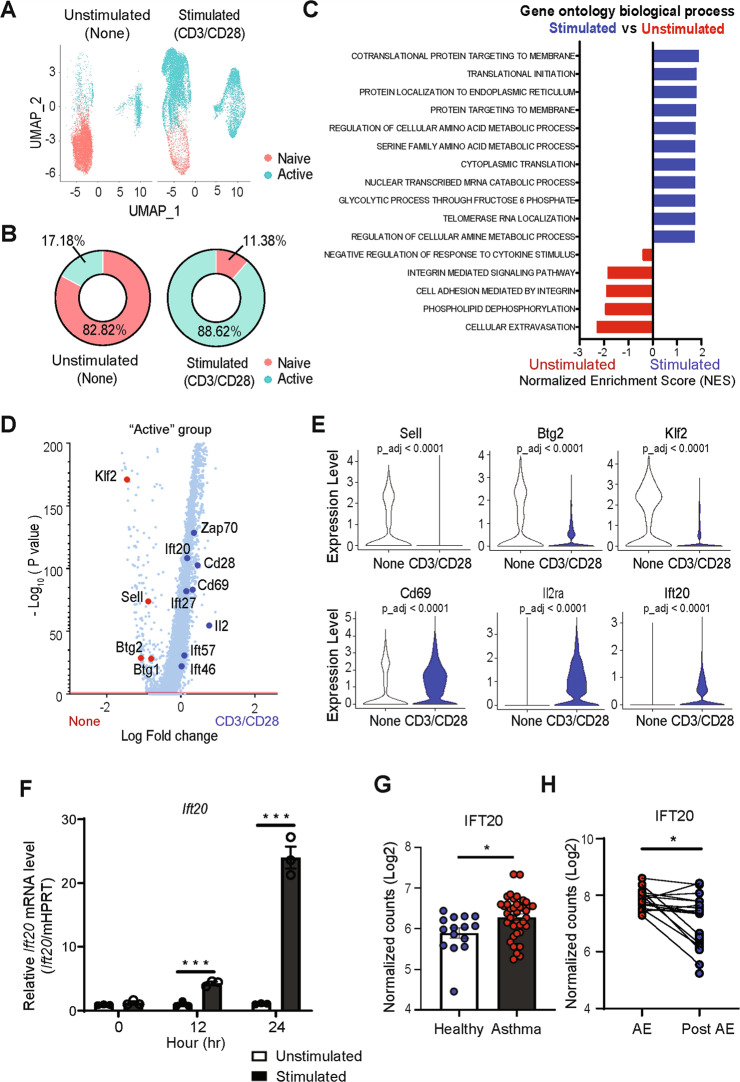
IFT20 is important for CD4^+^ T cell activation. **A–E** Analysis of single-cell RNA sequencing (scRNA-seq) data from CD4^+^ T cells either left unstimulated or stimulated with anti-CD3 and anti-CD28 antibodies. **A** Uniform manifold approximation and projection (UMAP) of unstimulated CD4^+^ T cells (none, 8,253 cells) and CD3/CD28-stimulated CD4^+^ T cells (CD3/CD28, 10,320 cells). The clusters were arranged into “naïve” and “active” groups based on the expression of *Sell* and *Cd69*. **B** Doughnut graphs depicting the percentages of cells in the “naïve” and “active” groups in unstimulated (none) and CD3/CD28-stimulated CD4^+^ T cells (CD3/CD28). **C** Gene set enrichment analysis (GSEA) using Gene Ontology biological process gene sets. The statistically significant gene set (FDR < 0.25) was sorted in order of the normalized enrichment score (NES). **D** Volcano plot showing differentially expressed genes (DEGs) in unstimulated (none) *vs*. CD3/CD28-stimulated CD4^+^ T cells (CD3/CD28). Each dot represents an individual gene. Significant DEGs in the none and CD3/CD28 groups are colored red and blue, respectively. **E** Violin plots for genes showing significant differential expression in the volcano plot. **F** Time course of *Ift20* expression in response to CD4^+^ T cell activation. The levels of *Ift20* mRNA were measured by quantitative reverse transcription (qRT)-PCR in CD4^+^ T cells activated for 0, 12, and 24 h. Naïve CD4^+^ T cells enriched by magnetic beads were activated with 1 μg/mL anti-CD3 and 1 μg/mL anti-CD28 antibodies. **G** *IFT20* expression in an RNA-seq dataset derived from Th2-enriched CD4^+^ T cells isolated from the peripheral blood of healthy subjects and asthma patients (Gene Expression Omnibus [GEO] accession number GSE75011). **H** *IFT20* expression in a microarray dataset derived from nasal lavage cells from asthma patients with acute exacerbation (AE) and in a postexacerbation state (Post-AE) (GEO accession number, GSE30326). The data are shown as the mean ± standard error or the mean (SEM). Statistical significance was calculated using the unpaired Student’s *t* test (**F**, **G**) or paired Student’s *t* test (**H**). *P < 0.05, ***P < 0.001

Using the Seurat algorithm [15], nine clusters of CD4^+^ T cells were identified by unsupervised hierarchical clustering, and these were visualized using the uniform manifold approximation and projection (UMAP) technique. The group of cells expressing L-selectin (*Sell)*, Kruppel-like Factor 2 (*Klf2)*, and interleukin-7 receptor-α (*Il7ra*), a naïve CD4^+^ T cell marker gene, was classified as “naïve” (Fig. 1A and Supplementary Fig. 1C, D), and the group expressing *Cd69, Il2ra, and Il2* was classified as “active” (Fig. 1A and Supplementary Fig. 1C, D). This clustering of “naïve” and “active” groups based on *Sell* and *Cd69* expression was further validated by flow cytometry. Similar frequencies of CD69^+^CD4^+^ T cells in flow cytometry and “active” cells in scRNA-seq were detected, confirming that our clustering was accurate (Fig. 1B and Supplementary Fig. 1A). Gene set enrichment analysis (GSEA) using the Gene Ontology database showed that genes related to protein localization to the endoplasmic reticulum and protein targeting to the membrane were upregulated in stimulated CD4^+^ T cells relative to unstimulated cells (Fig. 1C). Comparison of genes upregulated in “none” relative to the “CD3/CD28” group, which corresponds to “active” clusters, revealed that *Klf2*, B cell translocation gene 2 (*Btg2)*, and *Sell*, three known quiescence genes, were highly expressed in unstimulated cells (Fig. 1D, E). Conversely, the known CD4^+^ T cell effector genes *Cd69* and *Il2ra* were upregulated in “CD3/CD28” cells (Fig. 1D, E). Notably, we also detected significant upregulation of *Ift20*, which encodes a protein involved in cilia formation, in the “CD3/CD28” group (Fig. 1E), with upregulation of *Ift20* in stimulated CD4^+^ T cells further confirmed by quantitative reverse transcription (qRT)-PCR (Fig. 1F). The *Ift20* RNA expression level was altered by costimulation by CD28 and T cell receptor (TCR) signaling, including CD3 signaling (Supplementary Fig. 2). In addition, no significant difference was observed in *Ift20* RNA expression among T cell subsets, except for T helper (Th) 2 cells (Supplementary Fig. 2).

IFT20 was previously reported to localize to the cytoplasm in ciliated cells [7]. Here, to determine the localization of IFT20 in CD4^+^ T cells, we performed immunofluorescence staining in CD4^+^ T cells left unstimulated or stimulated with anti-CD3 and anti-CD28 antibodies. We found that IFT20 localized to the cytoplasm in both groups of CD4^+^ T cells (Supplementary Fig. 3A). However, IFT20 was localized to one point in the cytoplasm of stimulated CD4^+^ T cells (Supplementary Fig. 3A). To confirm the precise position of cytoplasmic IFT20, we performed immunogold labeling with transmission electron microscopy (TEM), which revealed that IFT20 localized to the Golgi apparatus and endosomes in immune cells from the mouse spleen (Supplementary Fig. 3B).

To further assess the in vivo relevance of our findings, we analyzed *IFT20* expression in existing transcriptomic datasets from human patients with asthma, which is a chronic respiratory disease characterized by variable expiratory airflow limitation. Notably, we found that the expression of *IFT20* was increased in CD4^+^ T cells from patients suffering from asthma relative to those from healthy subjects (Gene Expression Omnibus (GEO) accession number GSE75011) (Fig. 1G). In addition, asthma patients with acute exacerbation exhibited increased *IFT20* expression in nasal lavage cells compared to that in a postexacerbation state, which is a stable state in asthma patients (GEO accession number GSE30326) (Fig. 1H). These data indicate a possible association between the occurrence of asthma and elevated expression of IFT20. Therefore, we proceeded to investigate the role of IFT20 in asthma pathogenesis.

### Deficiency of IFT20 alleviates allergen-induced airway inflammation

To elucidate the role of IFT20 in CD4^+^ T cell activation in vivo, we generated CD4^+^ T cell-specific IFT20-KO mice using Cre-*lox* recombination technology (Supplementary Fig. 4A). To this end, *loxP* sites were inserted into introns 1 and 3 of the *Ift20* gene. We then bred these mice to animals expressing the *Cre* gene in CD4^+^ T cells, leading to removal of exons 2 and 3 from the *Ift20* gene via Cre-mediated recombination in CD4^+^ T cells (Supplementary Fig. 4B). We confirmed this specific reduction in IFT20 by performing western blot and qRT‒PCR analyses of CD4^+^ T cells enriched using magnetic beads. Our data indicated that IFT20 was absent only in CD4^+^ T cells in the conditional IFT20-KO mice (Supplementary Fig. 4C, D). This IFT20 deficiency in CD4^+^ T cells did not significantly affect T cell development or distribution to peripheral tissue (Supplementary Fig. 4E). Thus, the conditional IFT20-KO mice appeared similar to WT mice under normal conditions.

**Fig. 2:**
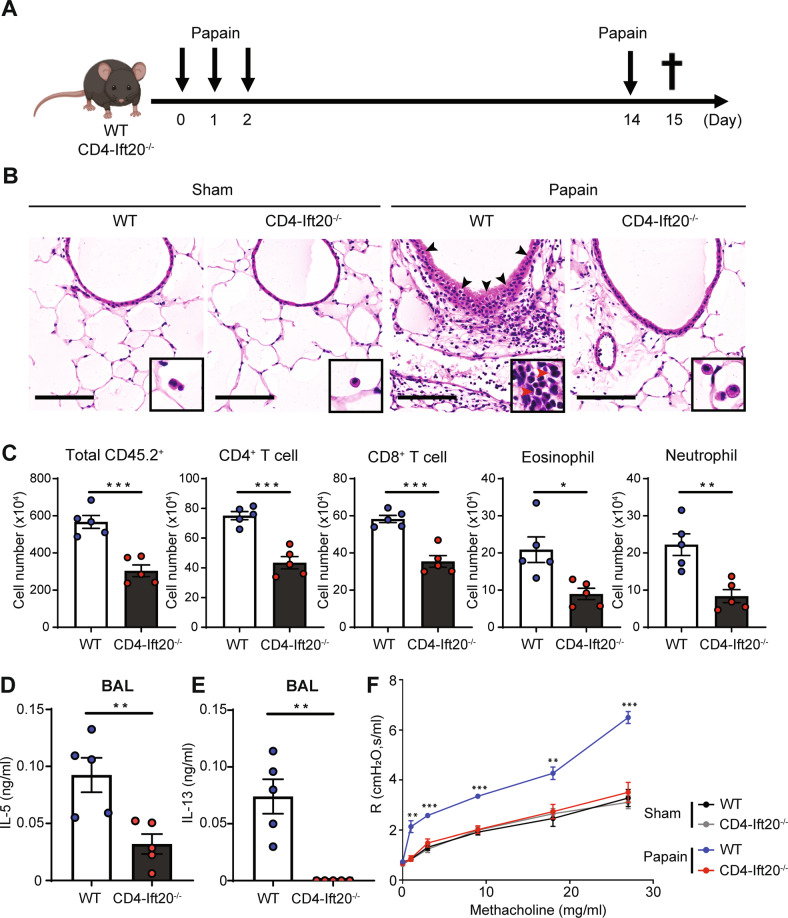
IFT20 deficiency alleviates protease-induced airway inflammation. **A** Overview of the papain-induced airway inflammation model. Mice were sensitized to a protease allergen by administration of 25 μg papain via intranasal injection for three consecutive days. The animals were then intranasally challenged with the same concentration of papain on Day 14 after the first intranasal injection, and lung tissue and bronchoalveolar lavage (BAL) fluid were harvested on Day 15. **B** Representative hematoxylin and eosin (H&E) staining images of lung tissue from papain-treated and sham control-treated WT and IFT20-KO mice. Black and red arrows indicate goblet cell hyperplasia and eosinophils, respectively. Scale bar, 100 μm. **C** Total numbers of CD45.2^+^ immune cells (*n* = 5), CD4^+^ T cells (*n* = 5), CD8^+^ T cells (*n* = 5), eosinophils (*n* = 5), and neutrophils (*n* = 5) in the lungs of papain-treated WT and IFT20-KO mice. Levels of (**D**) interleukin (IL)-5 (*n* = 5) and (**E**) IL-13 (*n* = 5) in BAL fluid from papain-treated WT and IFT20-KO mice were measured by enzyme-linked immunosorbent assay (ELISA) (*n* = 5). **F** Airway resistance to intratracheal methacholine in WT and IFT20-KO mice following administration of papain (*n* = 5). The representative images in (**B**) were obtained from more than two independent experiments. In **C**–**F**, the data shown represent more than three independent experiments. The data are shown as the mean ± SEM. Significance was calculated by unpaired Student’s *t* test. *P < 0.05, **P < 0.01, ***P < 0.001

We then assessed the effect of IFT20 on asthma induction by exposing conditional IFT20-KO mice and WT mice to papain according to a defined schedule that induces allergic inflammation (Fig. 2A). After 15 days of papain inhalation, lung tissue from IFT20-KO and WT mice was isolated and analyzed by H&E staining. Notably, we detected substantial goblet cell hyperplasia and infiltration of various immune cells that are hallmarks of airway inflammation in papain-treated WT mice but not in papain-treated IFT20-KO mice (Fig. 2B). To further identify these infiltrating immune cells, we performed flow cytometry analysis of cells from lung tissue. We observed reduced numbers of infiltrating total CD45.2^+^ immune cells as well as reduced numbers of CD4^+^ T cells, CD8^+^ T cells, eosinophils, and neutrophils in IFT20-KO mice relative to WT mice (Fig. 2C). We also detected decreased levels of interleukin (IL)-5, a cytokine required for eosinophil recruitment, and IL-13, which is essential for goblet cell hyperplasia, in bronchoalveolar lavage (BAL) fluid from IFT20-KO mice compared to WT mice (Fig. 2D, E). As a result, papain-treated IFT20-KO mice showed a reduction in airway hyperresponsiveness compared to that of papain-treated WT mice (Fig. 2F). IFT20 deficiency in CD4^+^ T cells reduced airway inflammation in OVA-induced and protease-induced asthma models. We observed decreased goblet cell hyperplasia and infiltration of various immune cells in OVA-treated WT mice but not in OVA-treated IFT20-KO mice (Supplementary Fig. 6B). OVA-treated IFT20-KO mice showed reduced numbers of CD45.2^+^ immune cells, eosinophils, and neutrophils relative to those of WT mice (Supplementary Fig. 6C). These data indicate that IFT20 deficiency alleviates airway inflammation in a mouse asthma model.

### IFT20 acts as a critical regulator of central supramolecular activation cluster formation

**Fig. 3:**
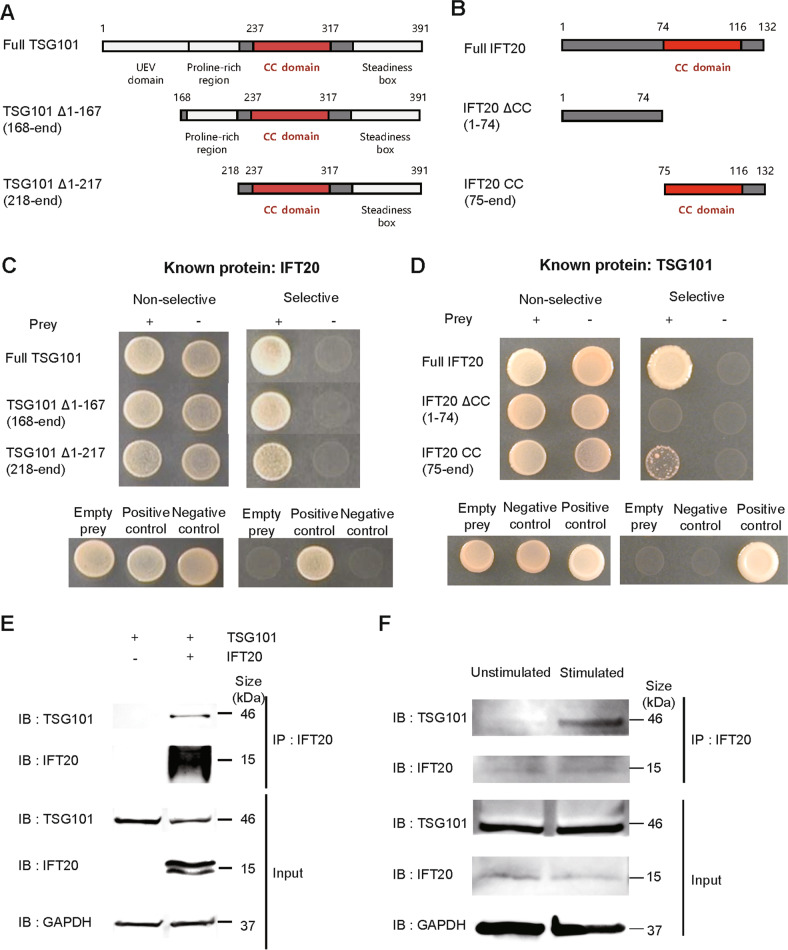
IFT20 and TSG101 interact and synergize with each other. **A** Schematic linear diagram of TSG101. **B** Schematic linear diagram of IFT20. **C** Yeast cells transformed with the IFT20 plasmid and prey plasmids containing potential IFT20-binding partners were cultured on nonselective (+histidine and adenosine) or selective SD media (–histidine and adenosine). The growth of yeast on selective media indicates the presence of protein‒protein interactions. **D** Yeast cells transformed with the TSG101 plasmid and prey plasmids containing potential IFT20-binding partners were cultured on nonselective (+histidine and adenosine) or selective SD medium (–histidine and adenosine). The growth of yeast on selective medium indicated the presence of protein‒protein interactions. **E** Coimmunoprecipitation of IFT20 and TSG101 from lysates of HEK293 T cells overexpressing the indicated proteins. **F** Coimmunoprecipitation of endogenous IFT20 and TSG101 proteins in Jurkat cell lysates. Jurkat cells were activated with 1 μg/mL anti-CD3 and 1 μg/mL anti-CD28 antibodies for 24 h. In **E–F**, the data represent more than three independent experiments. UEV ubiquitin E2 variant, CC coiled coil, IB immunoblot, IP immunoprecipitation

**Fig. 4:**
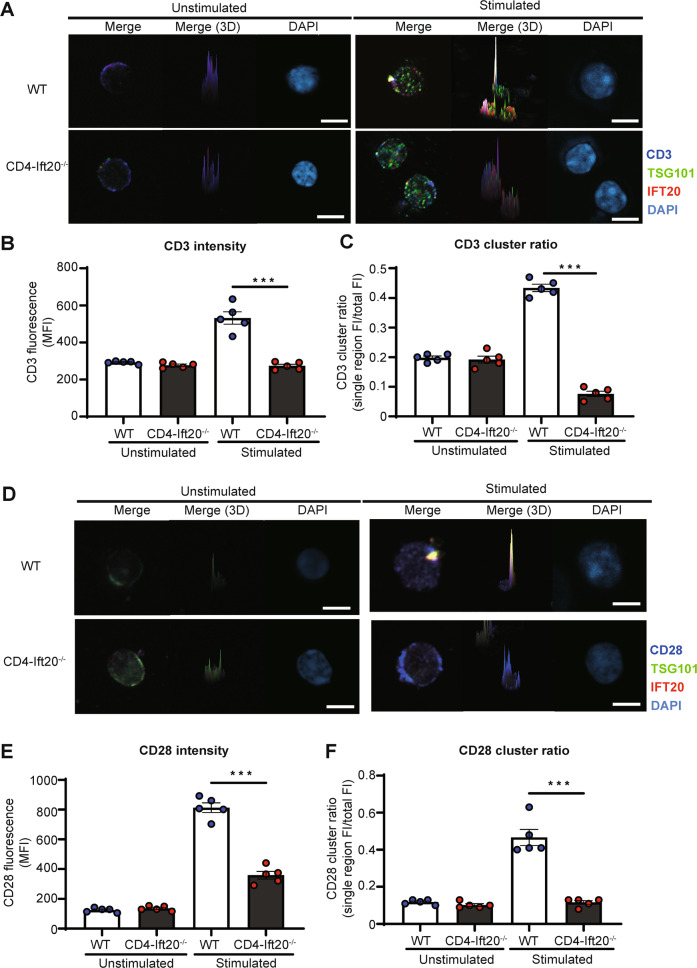
IFT20 acts as a critical regulator of c-SMAC formation. **A–F** Naïve CD4^+^ T cells from WT and IFT20-KO mice were enriched by magnetic beads and activated with 1 μg/mL anti-CD3 and 1 μg/mL anti-CD28 antibodies for 8 h (*n* = 5). **A** Representative immunofluorescence confocal microscopy images of unstimulated and stimulated CD4^+^ T cells from WT and IFT20-KO mice stained for CD3 (blue), IFT20 (red), TSG101 (green), and DAPI (light blue). Using ZEN 3.2 software, 3D images were obtained. Scale bar, 5 μm. CD3 intensities (**B**) and CD3 cluster ratios (**C**) were calculated with ZEN 3.2 software. **D** Representative immunofluorescence confocal microscopic images of unstimulated and stimulated CD4^+^ T cells from WT and IFT20-KO mice stained for CD28 (blue), IFT20 (red), TSG101 (green), and DAPI (light blue). 3D images were obtained with ZEN 3.2 software. Scale bar, 5 μm. CD28 intensities (**E**) and CD28 cluster ratios (**F**) were calculated with ZEN 3.2 software. The images in (**A**) and (**D**) represent five independent experiments. In **B**–**C** and **E**–**F**, the data represent more than three independent experiments. The data are shown as the mean ± SEM. Statistical significance was calculated by unpaired Student’s *t* test. ***P < 0.001

To further elucidate the detailed mechanism by which CD4^+^ T cell-specific IFT20 affects airway inflammation, we examined the role of IFT20 as a transporter protein. We hypothesized that the cargo protein carried by IFT20 might regulate the function of CD4^+^ T cells. To uncover proteins that interact with IFT20, we performed a yeast two-hybrid screen (Supplementary Fig. 7). We found that TSG101, a protein that recognizes ubiquitinated cargo and promotes endocytosis, was a binding partner of IFT20 (Fig. 3C). We constructed TSG101 mutants (Fig. 3A) and found that the site at which TGS101 bound to IFT20 contained a coiled-coil domain (Fig. 3C and Supplementary Fig. 8A). Next, we constructed IFT20 mutants to specifically determine the site of IFT20 that binds to TSG101 (Fig. 3B), which showed that TSG101 bound to the coiled-coil domain of IFT20 (Fig. 3D and Supplementary Fig. 8B). Using co-IP experiments, we confirmed the direct protein‒protein interaction between IFT20 and TSG101 in HEK293T cells (Fig. 3E). In addition, the interaction of IFT20 and TSG101 at an endogenous level was increased in in vitro-stimulated Jurkat cells relative to unstimulated Jurkat cells (Fig. 3F). To further validate this interaction in CD4^+^ T cells, we performed immunofluorescence staining with confocal microscopy. We observed increased IFT20 and TSG101 intensity and colocalization of IFT20 and TSG101 in CD4^+^ T cells stimulated with anti-CD3 and anti-CD28 antibodies (Fig. 4A, D, and Supplementary Fig. 9). These data indicate that the protein‒protein interaction between IFT20 and TSG101 is increased in a T cell activation-dependent manner.

Next, we hypothesized that the interaction between IFT20 and TSG101 might be associated with a change in CD4^+^ T cell function. We discovered that CD3 and CD28 were also concentrated at one point with IFT20 and TSG101 in stimulated WT CD4^+^ T cells (Fig. 4A, D). In addition, the intensity of CD3 and CD28 was higher in stimulated WT CD4^+ ^T cells than in unstimulated WT CD4^+^ T cells (Fig. 4B, E). The convergence of CD3 and CD28 in stimulated CD4^+^ T cells results in the formation of a central supramolecular activation cluster (c-SMAC), a complex that plays a role in amplifying CD4^+^ T cell activation [16–18]. To assess the degree of c-SMAC formation regulated by IFT20 and TSG101, we performed cluster ratio analysis. This approach assesses the proportion of the intensity in a specific region inside the cell relative to the total whole-cell intensity. As such, a high cluster ratio would indicate that c-SMAC formation is well established. Here, we observed decreased staining intensity for the c-SMAC components CD3 and CD28 in IFT20-KO CD4^+^ T cells (Fig. 4B, E), and the results from the cluster ratio analysis were consistent with disruption of c-SMAC formation in these cells (Fig. 4C, F). These data suggest that the TSG101–IFT20 axis may converge on CD3 and CD28, leading to c-SMAC formation. Thus, we speculate that ubiquitinated proteins (e.g., CD3 and CD28) may be recognized by TSG101 and transported via IFT20 to form c-SMAC.

### IFT20 enhances AKT-mTOR signaling and CD4^+^ T-cell proliferation

**Fig. 5:**
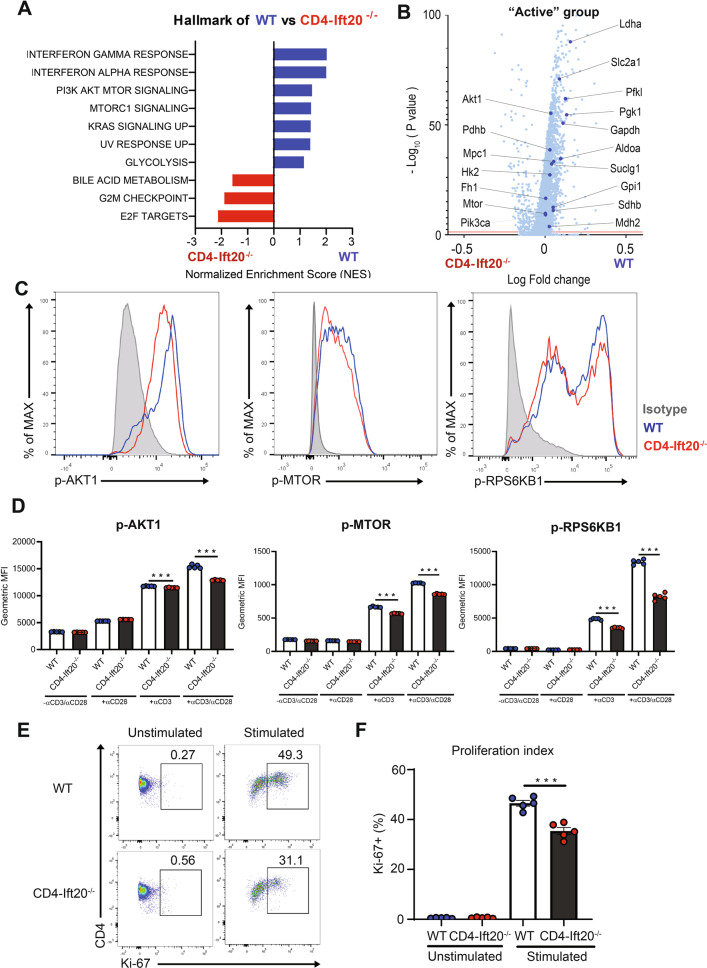
IFT20 enhances AKT–mTOR signaling and CD4^+^ T cell proliferation. **A**, **B** Naïve CD4^+^ T cells from WT and IFT20-KO mice were activated with anti-CD3 and anti-CD28 antibodies for 24 h and analyzed by scRNA-seq. **A** GSEA using hallmark gene sets. The statistically significant gene set (FDR < 0.25) was sorted in order of the normalized enrichment score (NES). **B** Volcano plot showing DEGs in CD4^+^ T cells from WT and IFT20-KO mice. Each dot represents an individual gene. Significant DEGs in the WT group are colored blue. **C–F** Naïve CD4^+^ T cells from IFT20-KO and WT mice were enriched by magnetic beads and activated with 1 μg/mL anti-CD3 and 1 μg/mL anti-CD28 antibodies for 2 days. **C** The levels of phosphorylated AKT1 (p-AKT1), mTOR (p-mTOR), and RPS6KB1 (p-RPS6KB1) were assessed by flow cytometry using phospho-specific antibodies. **D** Bar graphs indicating the mean fluorescence intensity (MFI) of p-AKT1, p-MTOR, and p-RPS6KB1 expression in IFT20-KO and WT cells. **E** Ki-67 staining frequencies were examined by flow cytometry. **F** Bar graphs indicating the frequencies of Ki-67 staining. The data are shown as the mean ± SEM. Statistical significance was calculated by unpaired Student’s *t* test. ***P < 0.001

Next, we investigated which signaling pathway in CD4^+^ T cells may be involved in regulating c-SMAC formation with IFT20. Therefore, we performed scRNA-seq on WT and IFT20-deficient stimulated CD4^+^ T cells. Interestingly, GSEA using a hallmark gene set revealed that the phosphoinositide 3-kinase alpha (PI3K)-AKT–mechanistic target of rapamycin (mTOR) signaling cascade was upregulated in stimulated WT CD4^+^ T cells relative to IFT20-deficient CD4^+^ T cells (Fig. 5A). Conversely, we observed decreased AKT-mTOR-related gene expression (e.g., *Pik3ca*, *Akt1*, and *Mtor*) in cells from IFT20-KO mice in a volcano plot of the data (Fig. 5B and Supplementary Fig. 10A). To identify whether IFT20 is involved in the phosphorylation of AKT and mTOR, we performed intracellular staining with phosphor-specific antibodies against AKT1, mTOR, and ribosomal protein S6 kinase B1 (RPS6KB1), a downstream protein activated by AKT-mTOR signaling (Fig. 5C). Phosphorylation of AKT1, mTOR, and RPS6KB1 was reduced in IFT20-deficient CD4^+^ T cells relative to WT CD4^+^ T cells (Fig. 5D). Because CD4^+^ T-cell proliferation has been reported to be regulated by activation of AKT–mTOR signaling [19], we conducted Ki-67 staining to analyze the effect of IFT20 deficiency on CD4^+^ T-cell proliferation (Fig. 5E). We detected a decreased frequency of Ki-67 staining in CD4^+^ T cells from IFT20-KO mice relative to those from WT mice (Fig. 5F). Thus, our data indicate that IFT20 enhances the AKT–mTOR signaling pathways and promotes CD4^+^ T cell proliferation.

### IFT20 regulates aerobic glycolysis and cellular respiration

T-cell activation and differentiation have been reported to be regulated by various metabolic pathways [20]. In particular, there is emerging evidence that naïve T cells acquire adenosine triphosphate (ATP) through OXPHOS in the mitochondria, whereas differentiation into Th1, Th2, and Th17 cells requires energy from aerobic glycolysis [21]. To our knowledge, there are no published reports describing a role for IFT20 in glucose metabolism. Therefore, we investigated this possibility by analyzing our scRNA-seq data from stimulated CD4^+^ T cells and found that glycolysis-related genes were upregulated in CD4^+^ T cells of WT mice compared to those from IFT20-KO mice (Fig. 5A). Conversely, we found that numerous glycolysis-related genes, including *solute carrier family 2 member 1* (*Slc2a1*), *glucose-6-phosphate isomerase* 1 (*Gpi1*), *phosphoglycerate kinase 1* (*Pgk1*), and *lactate dehydrogenase A* (*Ldha*), were significantly downregulated in CD4^+^ T cells from IFT20-KO mice (Fig. 5B and Supplementary Fig. 10B).

**Fig. 6:**
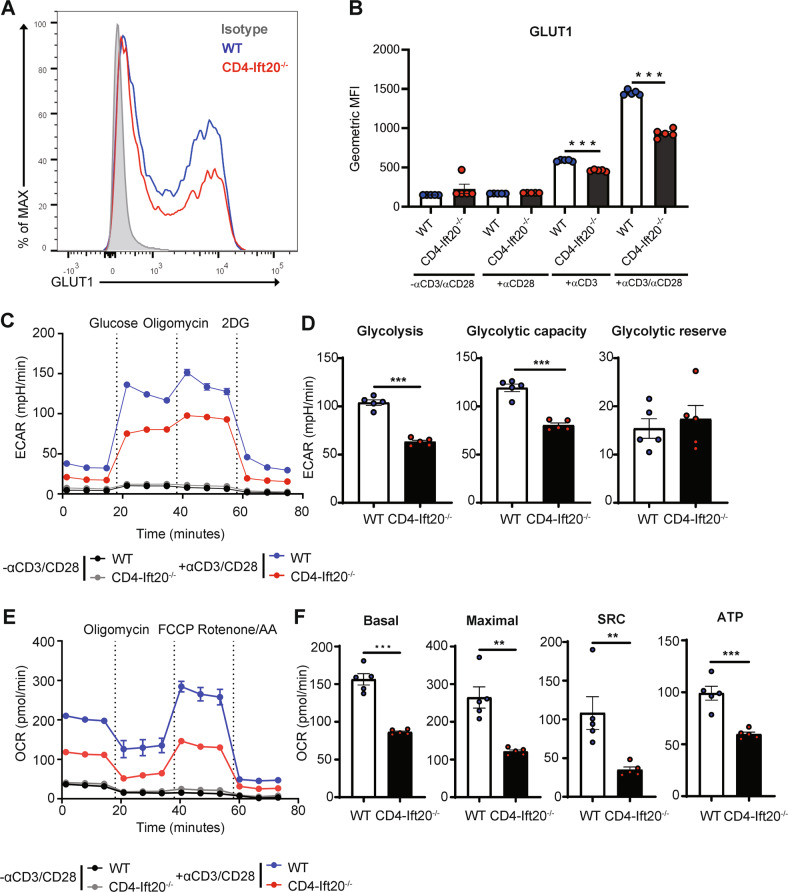
IFT20 regulates aerobic glycolysis and cellular respiration in CD4^+^ T cells. **A**, **B** Naïve CD4^+^ T cells from IFT20-KO and WT mice enriched by magnetic beads were activated with 1 μg/mL anti-CD3 and 1 μg/mL anti-CD28 antibodies for 24 h. **A** The levels of GLUT1 were examined by flow cytometry. Representative MFI values of GLUT1 expression in IFT20-KO and WT cells are shown. **B** Bar graphs indicating the MFIs of GLUT1 levels are shown. **C–F** Naïve CD4^+^ T cells from IFT20-KO and WT mice enriched by magnetic beads were activated with 1 μg/mL anti-CD3 and 1 μg/mL anti-CD28 antibodies for 48 h. **C** Extracellular acidification rate (ECAR) in CD4^+^ T cells from WT (*n* = 5) and IFT20-KO (*n* = 5) either left unstimulated or stimulated with 1 μg/mL anti-CD3 and 1 μg/mL anti-CD28 antibodies for 48 h (*n* = 5) was measured by the glycolysis stress test. **D** Glycolysis, glycolytic capacity and glycolytic reserve of CD4^+^ T cells from WT and IFT20-KO mice activated as in (**C**) (*n* = 5). **E** The oxygen consumption rate (OCR) was measured by the mitochondrial stress test in unstimulated (*n* = 5) and stimulated (*n* = 5) CD4^+^ T cells from WT and IFT20-KO mice. **F** Basal respiration, maximal respiration, spare respiratory capacity, and ATP production of stimulated CD4^+^ T cells from WT and IFT20-KO mice (*n* = 5). SRC, Spare respiratory capacity; ATP, ATP production. The data are shown as the mean ± SEM. Statistical significance was calculated by unpaired Student’s *t* test. **P < 0.01, ***P < 0.001

Based on these findings, we next examined the effect of IFT20 deficiency on overall glucose metabolism in CD4^+^ T cells. When these cells are activated, glycolysis is carried out using external glucose that is taken up via GLUT1, the upstream transporter for the glycolysis pathway [22]. Since the expression of *Slc2a1*, a coding gene of GLUT1, was downregulated in IFT20-deficient CD4^+^ T cells, we hypothesized that IFT20 might be involved in glucose uptake. When we measured the levels of GLUT1 in CD4^+^ T cells from WT and IFT20-KO mice, we found that GLUT1 was decreased in CD4^+^ T cells from IFT20-KO mice (Fig. 6A, B). To determine whether glycolysis is affected by IFT20 at the mRNA and protein levels, we performed glycolysis stress tests on stimulated CD4^+^ T cells from WT and IFT20-KO mice using an XFe96 Extracellular Flux Analyzer (Fig. 6C). We detected a reduced ECAR in stimulated CD4^+^ T cells from IFT20-KO mice compared to those from WT animals (Fig. 6C). We then measured the cellular levels of glycolysis, glycolytic capacity (i.e., ability to obtain the maximum ATP from glycolysis), and glycolytic reserve. We found that CD4^+^ T cells from IFT20-KO mice displayed lower glycolysis and glycolytic capacity than WT CD4^+^ T cells (Fig. 6D). However, we observed no difference in glycolytic reserve between stimulated IFT20-deficient CD4^+^ T cells and WT CD4^+^ T cells (Fig. 6D). These data indicated that glycolysis was decreased in response to IFT20 deficiency.

In addition to regulation of glycolysis-related genes by IFT20, we found reduced expression of genes involved in pyruvate metabolism (e.g., *mitochondrial pyruvate carrier 1 (Mpc1)* and *pyruvate dehydrogenase E1 subunit beta (Pdhb)*) and the TCA cycle (e.g., *succinate-CoA ligase GDP/ADP-forming subunit alpha (Suclg1)*, *fumarate hydratase 1 (Fh1)*, and *malate dehydrogenase 2 (Mdh2)*) in CD4^+^ T cells from IFT20-KO mice (Fig. 5B and Supplementary Fig. 10C, D). These results suggest that abnormalities in pyruvate metabolism and the TCA cycle can lead to dysfunction of cellular respiration. To confirm the involvement of IFT20 in the regulation of cellular respiration, we performed mitochondrial stress tests in stimulated CD4^+^ T cells from WT and IFT20-KO mice using an XFe96 Extracellular Flux Analyzer (Fig. 6E). We detected a decreased OCR in stimulated CD4^+^ T cells from IFT20-KO mice compared to those from WT animals (Fig. 6E). We then measured the cellular levels of OXPHOS parameters, including basal respiration, maximal respiration, spare respiratory capacity (i.e., ability to cover sudden increased energy needs through mitochondrial OXPHOS), and ATP production. We found that spare respiratory capacity, ATP production, and both basal and maximal respiration were reduced in CD4^+^ T cells from IFT20-KO mice (Fig. 6F). These data indicated that both OXPHOS and cellular respiration were decreased in response to IFT20 deficiency.

We therefore hypothesized that mitochondrial dysfunction may be present in CD4^+^ T cells from IFT20-KO mice. To test this hypothesis, we stimulated naïve CD4^+^ T cells from WT and IFT20-KO mice with anti-CD3 and anti-CD28 antibodies and measured the mitochondrial membrane potential of living cells in each group using MitoTracker^TM^ Red CMXRos. We found that CD4^+^ T cells from IFT20-KO mice contained small mitochondria that were fragmented into several pieces (Supplementary Fig. 11A). In addition, the overall mitochondrial membrane potential was reduced in these cells (Supplementary Fig. 11B). Thus, our data revealed impaired mitochondrial function in CD4^+^ T cells from IFT20-KO mice. Overall, our findings showed that IFT20 deficiency in CD4^+^ T cells inhibited the expression of key glycolytic enzymes and cellular GLUT1 levels, resulting in decreased glucose uptake that attenuated both glycolysis and OXPHOS, leading to mitochondrial dysfunction in CD4^+^ T cells from IFT20-KO mice.

### Deficiency of TSG101 reduces activation of CD4^+^ T cells

The role of TSG101 in CD4^+^ T cell activation is controversial. One study has found that TSG101 deficiency increases calcium influx and induces chronic TCR signaling [23]. However, another study by the same group has reported that TSG101 is important for the formation of microvesicles in endosomes and that TSG101 deficiency leads to reduced TCR signaling via PLCγ regulation [24]. Notably, a critical limitation of these two studies is that the intensity of TCR signaling was measured from images using an indirect method. To elucidate the role of TSG101 in CD4^+^ T cell activation, we generated CD4^+^ T cell-specific TSG101-KO mice using Cre-*lox* recombination technology (Supplementary Fig. 12A). To this end, *loxP* sites were inserted into introns 1 and 2 of the *Tsg101* gene. We then bred these mice to animals expressing the *Cre* gene in CD4^+^ T cells, leading to removal of exon 1 from the *Tsg101* gene via Cre-mediated recombination in CD4^+^ T cells (Supplementary Fig. 12B). CD4^+^ T cell-specific TSG101 deficiency under normal conditions led to decreased CD4^+^ T cell and CD8^+^ T cell numbers and increased γδ T cell numbers in the spleen. The remaining T cells in the spleen of TSG101-KO mice showed increased CD44 expression compared to that of WT mice. B cells were not affected by TSG101 deficiency (Supplementary Fig. 12C).

**Fig. 7:**
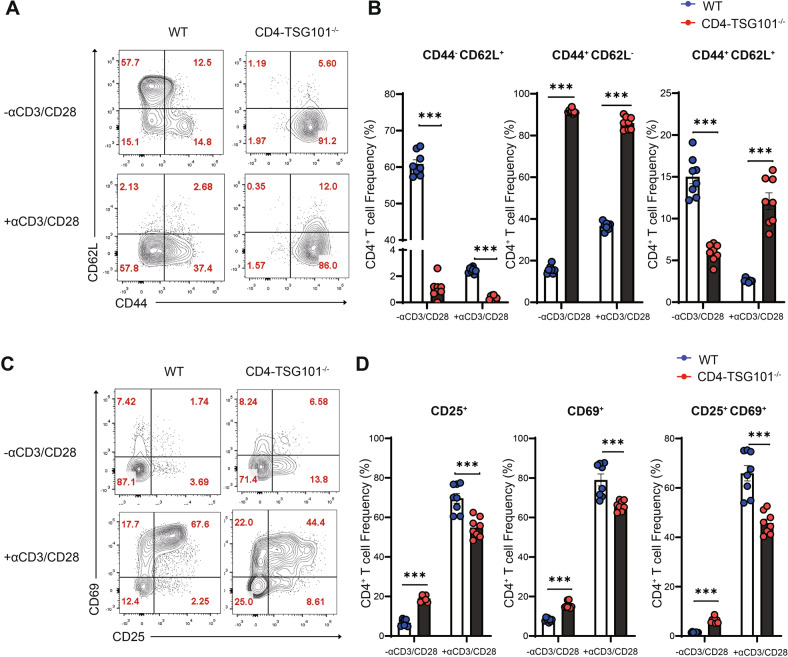
TSG101 deficiency reduced CD4^+^ T cell activation. **A**–**D** CD4^+^ T cells from the spleens of TSG101-KO and WT mice were enriched by magnetic beads and stimulated with 1 μg/mL anti-CD3 and 1 μg/mL anti-CD28 antibodies for 8 h. **A** Representative flow cytometry plots showing the expression of CD44 and CD62L in unstimulated and stimulated CD4^+^ T cells from WT and TSG101-KO mice. **B** The frequencies of CD44^-^CD62L^+^, CD44^+^CD62L^-^, and CD44^+^CD62L^+^ T cells were measured by flow cytometry (*n* = 8). **C** Representative flow cytometry plots showing the expression of CD25 and CD69 in unstimulated and stimulated CD4^+^ T cells from WT and IFT20-KO mice. **D** The frequencies of CD25^+^(CD25^+^CD69^+^ and CD25^+^CD69^-^), CD69^+^(CD25^+^CD69^+^ and CD25^-^CD69^+^), and CD25^+^CD69^+^ T cells were measured by flow cytometry (*n* = 8). In **A**–**D**, the data represent more than three independent experiments. CD4^+^ T cells were obtained by negative selection using a CD4^+^ T cell enrichment kit. The data are expressed as the mean ± SEM. Significance was calculated using an unpaired Student’s *t* test. ***P < 0.001

Using magnetic bead-sorted CD4^+^ T cells from the spleens of TSG101-KO mice and WT mice, we obtained CD4^+^  T cells activated in vitro with anti-CD3 and anti-CD28 antibodies. Before stimulation, TSG101-deficient CD4^+^ T cells lacked naïve (CD44^-^CD62L^+^) T cells and had a significantly decreased number of central memory (CD44^+^CD62L^+^) T cells, but effector memory (CD44^+^CD62L^-^) T cell numbers were significantly increased compared with those of WT CD4^+^ T cells. Upon activation, T cells lacking TSG101 showed reduced colocalization of IFT20 with CD3 molecules on the cell surface, indicating that TSG101 is required for the binding of IFT20 to the TCR complex (Supplementary Fig. 14). TSG101-deficient CD4^+^ T cells showed fewer naïve CD4^+^ T cells (CD44^-^CD62L^+^) but more effector memory (CD44^+^CD62L^-^) and central memory (CD44^+^CD62L^+^) CD4^+^ T cells than WT CD4^+^ T cells (Fig. 7A, B). However, these increased numbers of central memory and effector memory CD4^+^ T cells among TSG101-deficient CD4^+^ T cells showed lower expression of CD25 and CD69, which are activation markers of CD4^+^ T cells, than WT CD4^+^ T cells (Fig. 7C, D). Reduced expression of CD25 and CD69 was also observed in IFT20-deficient CD4^+^ T cells (Supplementary Fig. 13A, B). These results suggest that IFT20 and TSG101 play an important role in CD4^+^ T-cell activation.

## Discussion

In this study, we revealed that TSG101 binds to the coiled-coil domain of IFT20. By generating conditional TSG101-KO and conditional IFT20-KO mice, we identified that the protein‒protein interaction between IFT20 and TSG101 plays an important role in CD4^+^ T cell activation by regulating c-SMAC formation and metabolism, which is involved in processes such as aerobic glycolysis and cellular respiration. In particular, using two murine models of allergen-induced asthma, we demonstrated that CD4^+^ T cell-specific IFT20 deficiency leads not only to reduced numbers of lung-infiltrating immune cells, including eosinophils and neutrophils, but also to decreased airway hyperresponsiveness relative to that of WT mice.

Previous studies on IFT proteins that were conducted in the flagella of *Chlamydomonas reinhardtii* have focused on the functions of these proteins in ciliated tissues and cells, such as the those in the retinas, kidneys, and sperm [25, 26]. Therefore, research on the role of IFT20 in immune cells, a nonciliated cell type, is relatively limited. According to Finetti et al. IFT20 is present in immune cells, such as lymphoid and myeloid cells, and is mainly responsible for TCR/CD3 trafficking [27]. When the antigen peptide of MHC class II on APCs binds to the TCR, various TCRs distributed on the surface of a T cell accumulate for c-SMAC formation [27]. IFT20 colocalizes with endosome-associated proteins, including Rab4, Rab8, and Rab11, to transport ubiquitinated TCR [28, 29]. However, the mechanism by which IFT20 initially interacts with ubiquitinated TCRs has not been elucidated. Here, we report for the first time that the coiled-coil domain of IFT20 interacts directly with TSG101. Localized to early endosomes, the multivesicular body, and lysosomes, TSG101 has been shown to play a central role in the recruitment of ubiquitinated proteins into endosomes [30, 31]. Although the precise role of TSG101 in immune cells is not well understood, several studies have shown that TSG101 recognizes ubiquitinated TCR and is responsible for the initiation of endosome formation to transport ubiquitinated TCR [23, 24]. Based on previous research, we suggest that early c-SMAC formation begins with the binding of IFT20 to TSG101, which might be involved in the endocytosis of ubiquitinated TCRs.

We first generated CD4^+^ T cell-specific conditional TSG101-KO mice. In these mice, B cells were not affected under normal conditions, but CD4^+^ and CD8^+^ T cells were decreased. Interestingly, most T cells observed in the spleens of unstimulated TSG101-KO mice were not naïve T cells but were effector memory T cells, indicating that naïve TSG101-deficient T cells are susceptible to apoptosis. Some data suggest that TSG101 prevents apoptosis caused by cell stresses, such as DNA damage and oxidative damage, and that depletion of TSG101 promotes increased cellular damage via lysosome malfunctions [32]. TSG101-deficient cells are vulnerable to DNA damage, resulting in apoptosis [33]. In addition, upregulation of TSG101 protects against oxidative stress, while downregulation of TSG101 aggravates reactive oxygen species-induced damage [34]. Compared with naïve T cells, memory T cells can tolerate cell stress and are resistant to apoptosis [35, 36]. Taken together, these data suggest that naïve T cells are susceptible to cell stress, eventually undergoing apoptosis, and that only effector memory cells appear to survive among TSG101-deficient T cells.

Notably, we found that IFT20 enhances the phosphorylation of AKT-mTOR signaling molecules by forming a complex with TSG101 to promote c-SMAC formation. Previous studies have demonstrated the amplification of TCR/CD3 signaling by IFT20 in the Jurkat cell line [27, 37]. Hypothetically, a sequential increase in AKT-mTOR signaling may be expected due to the amplification of TCR/CD3 signaling by IFT20. However, activation of AKT-mTOR signaling is dependent on CD28 signaling rather than TCR/CD3 signaling [19, 38] Zheng et al. reported that T-cell anergy without costimulatory molecules such as CD28 reduced the phosphorylation of AKT-mTOR signaling compared to that achieved with full T-cell activation with costimulation, resulting in downregulation of the amino acid transporter CD98 and the transferrin receptor CD71 [39]. In our immunofluorescence imaging data, IFT20 was involved in the formation of c-SMACs induced by CD3 and CD28 stimulation. This is the first report to show the involvement of IFT20 in c-SMAC formation induced by costimulation with CD3 and CD28. We suggest that this amplification via costimulation by IFT20 results in enhanced AKT-mTOR signaling.

Glucose metabolism is closely linked to CD4^+^ T cell activation and maintenance of function [40]. In particular, CD4^+^ T cell activation requires metabolic reprogramming from OXPHOS to aerobic glycolysis [41], and effector CD4^+^ T cells enhance glucose uptake, an important first step in glycolysis, through upregulation of the glucose transporter GLUT1 [42]. However, the function of IFT20 in glucose metabolism has not been reported. We are the first to reveal that IFT20 regulates GLUT1 expression, as IFT20 deficiency leads to a reduction in GLUT1 levels, resulting in decreased glycolysis. In addition, we show that IFT20 deficiency reduces basal and maximal cellular respiration, as well as spare respiratory capacity, indicating that IFT20 also modulates cellular respiration via regulation of glucose metabolism. Furthermore, decreased glucose metabolites in cells lacking IFT20 exhibit mitochondrial dysfunction and reduced cellular respiration. Thus, we show that IFT20 controls glucose metabolism by regulating the expression of GLUT1.

Unlike naïve CD4^+^ T cells, which show reduced levels of GLUT1, effector CD4^+^ T cells stimulated by a TCR have increased levels of GLUT1, resulting in increased glucose uptake [43]. This increase in the surface expression of GLUT1 is triggered by AKT–mTOR signaling [44] and is inhibited by treatment with rapamycin, an mTOR inhibitor [45]. Here, we show that IFT20 induces c-SMAC formation by binding to TSG101, which leads to amplification of AKT–mTOR signaling. Consistent with this finding, IFT20 deficiency reduced AKT-mTOR signaling due to c-SMAC malformation. Thus, we propose that disruption of AKT-mTOR signaling resulting from IFT20 deficiency leads to reduced surface expression levels of GLUT1 in CD4^+^ T cells.

When exposed to various allergens, asthma patients experience coughing, wheezing, and dyspnea [46]. Although some patients can be treated with commercially available drugs, some experience severe refractory asthma and do not respond to these existing therapeutics [47]. Notably, prevalence rates for severe refractory asthma are increasing worldwide, thus increasing the burden on health care systems. Moreover, patients with this condition incur medical costs that are significantly higher than those with ordinary asthma [48, 49]. Severe refractory asthma is typically caused by smoking, fine dust, and ozone rather than by allergens [50, 51]. These substances induce direct damage to lung epithelial cells that, in turn, secrete alarmins such as IL-33, which stimulate innate lymphoid cells [52]. Patients with severe refractory asthma exhibit distinct pathophysiological features, including the presence of neutrophils and eosinophils, in their sputum [53]. Here, our flow cytometric analysis of mouse lung tissue from our allergen-induced asthma models confirmed increased levels of both eosinophils and neutrophils, consistent with observations in severe refractory asthma patients. Furthermore, IFT20 deficiency led to decreased levels of infiltrating eosinophils and neutrophils and reductions in inflammatory marker levels. Thus, our results suggest that the development of asthma drugs targeting IFT20 may represent a possible strategy for treating severe refractory asthma patients.

## Supplementary information

Supplementary tables

Supplementary figures

## Data Availability

All data are available in the main text or the supplementary appendix. The single-cell RNA sequencing data were deposited in GEO (GSE179622 and GSE179623) at the NCBI.
