# Supplementary material for: Regulation of c-SMAC formation and AKT-mTOR signaling by the TSG101-IFT20 axis in CD4+ T cells
Source: Cell Mol Immunol. 2023 Apr 7;20(5):525–39. doi: 10.1038/s41423-023-01008-x (PMC10202954; doi:10.1038/s41423-023-01008-x)
Supplement: Supplementary file 1 — Supplementary tables [file 41423_2023_1008_MOESM1_ESM.pdf]

Supplementary Table 1. Lists for flow cytometric antibody

Name	Clone	Color	Company	Catalog number
CD3	17A2	FITC	BioLegend	100204
CD4	GK1.5	FITC	BioLegend	100406
CD19	1D3	FITC	BD	561740
Ki-67	16A8	FITC	BioLegend	652410
NK1.1	PK136	FITC	BD	553164
IgG2aκisotype	eBR2a	FITC	Invitrogen	11-4321-82
CD3	145-2C11	PE	BioLegend	100308
CD44	IM7	PE	BD	553134
p-mTOR	MRRBY	PE	Invitrogen	12-9718-42
p- RPS6KB1	Cupk43k	PE	Invitrogen	12-9007-42
IgG1κisotype	MOPC-21	PE	BD	555749
IgG2aκisotype	RTK2758	PE	BioLegend	400508
CD44	IM7	PerCP-Cy5.5	BioLegend	103031
Ly6G	1A8	PerCP-Cy5.5	BD	560602
NK1.1	PK136	PerCP-Cy5.5	BioLegend	108727
CD4	GK1.5	APC	BioLegend	100412
CD25	7D4	APC	Miltenyi Biotec	130-102-787
CD62L	MEL-14	APC	BD	553152
CD69	H1.2F3	APC	BioLegend	104514
TCR γ/δ	GL3	APC	BioLegend	118116
Ly6C	HK1.4	APC	BioLegend	128016
p-AKT1	SDRNR	APC	Invitrogen	17-9715-42
IgG1κisotype	RTK2071	APC	BioLegend	400412
IgG2aκisotype	RTK2758	APC	BioLegend	400511
CD11c	N418	PECy7	BioLegend	117318
CD25	PC61	PECy7	BD	561780
CD8	53-6.7	APCCy7	BioLegend	100714
I-A/I-E	M5/114.15.2	APCCy7	BioLegend	107627
GLUT1	EPR3915	AF647	Abcam	Ab195020
CD3	17A2	AF700	BioLegend	100215
CD45.2	104	AF700	BD	560693
CD4	GK1.5	BV421	Invitrogen	48-0041-82
SiglecF	E50-2440	BV421	BD	562681
CD8	53-6.7	BV510	BD	563068
CD11b	M1/70	BV510	BD	562950
CD44	IM7	BV510	BD	563114
MitoTracker			Invitrogen	M7512
7-AAD			BioLegend	420404
Fixable viability stain 450			BD	562247
PI			Invitrogen	00-6990-50

Supplementary Table 2. Lists for western blot and Co-IP antibody

Name	Clone	Company	Catalog number
IFT20	Polyclonal	Proteintech	13615-1-AP
TSG101	4A10	Abcam	Ab83
β-actin	C4	Santa Cruz Biotech	SC-47778
GAPDH	14C10	Cell signaling	2118S
StarBright Blue 700 Goat Anti-Rabbit IgG		Bio-Rad	12004161
StarBright Blue 700 Goat Anti-Mouse IgG		Bio-Rad	12004158

Supplementary Table 3. Lists for ELISA antibody

Name	Clone	Company	Catalog number
Rat anti-Mouse IL-5	TRFK5	BD	554581
Biotin anti-Mouse IL-5	TRFK4	BD	554397
Rat anti-Mouse IL-13	eBioa13A	Invitrogen	14-7133-81
Biotin anti-Mouse IL-13	eBio1316H	Invitrogen	13-7135-85
Rat anti-Mouse IL-17A	TC11-18H10	BD	555068
Biotin anti-Mouse IL-17A	TC11-8H4	BD	555067
Rat anti-Mouse IFN-γ	R4-6A2	BD	551216
Biotin anti-Mouse IFN-γ	R4-6A2	BD	551506
HRP Streptavidin		BioLegend	405210

Supplementary Table 4. Lists for confocal and TEM antibody

Name	Clone	Company	Catalog number
IFT20	Polyclonal	Proteintech	13615-1-AP
TSG101	4A10	Abcam	Ab83
MitoTracker RedCMXRos		Invitrogen	M7512
CD3		Invitrogen	16-0031-85
CD28	145-2C11	BioLegend	102102
AF488 Goat Anti-Mouse IgG (H+L)	37.51	Jackson ImmunoResearch	115-545-166
AF594 Goat Anti-Rabbit IgG (H+L)	Polyclonal	Jackson ImmunoResearch	111-585-144
AF647 Goat Anti-Armenian Hamster IgG (H+L)	Polyclonal	Jackson ImmunoResearch	127-605-160
Goat-anti-Rabbit IgG (H&L)		Aurion	810.011
Mounting Medium with DAPI		Abcam	Ab104139

Supplementary Table 5. Lists for T cell differentiation antibody

Name	Clone	Company	Catalog number
CD3		Invitrogen	16-0031-85
CD28	145-2C11	BioLegend	102102
Anti-IL-4	11B11	BioXCell	BE0045
Anti-IFN-γ	XMG1.2	BioXCell	BE0055
IL-2		Biolegend	589104
IL-4		Peprotech	214-14
IL-6		Peprotech	216-16
IL-12		Peprotech	210-12
TGF-β1		Biolegend	580702
