# Supplementary material for: Regulation of c-SMAC formation and AKT-mTOR signaling by the TSG101-IFT20 axis in CD4+ T cells
Source: Cell Mol Immunol. 2023 Apr 7;20(5):525–39. doi: 10.1038/s41423-023-01008-x (PMC10202954; doi:10.1038/s41423-023-01008-x)
Supplement: Supplementary file 2 — Supplementary figures [file 41423_2023_1008_MOESM2_ESM.pdf]

Title: Regulation of c-SMAC formation and AKT-mTOR signaling by the TSG101-IFT20 axis in CD4+ T cells

Authors: Jiung Jeong[1,2], In Kang[1], Yumin Kim[3], Keun Bon Ku[1,4], Chae Won Kim[1], Jang Hyun Park[1], Hyun-Jin Kim[1], Jeongwoo La[1], Hi Eun Jung[1], Hyeon Cheol Kim[1], Young Joon Choi[1], Jaeho Kim[1], Joon Kim[1], Heung Kyu Lee[1,3]*

[1]Graduate School of Medical Science and Engineering, Korea Advanced Institute of Science and Technology (KAIST), Daejeon, 34141, Republic of Korea

[2]Department of Internal Medicine, Seoul National University Hospital, Seoul, 03080, Republic of Korea

[3]Department of Biological Sciences, Korea Advanced Institute of Science and Technology (KAIST), Daejeon, 34141, Republic of Korea

[4] Department of Convergent Research of Emerging Virus Infection, Korea Research Institute of Chemical Technology, Daejeon, 34141, Republic of Korea

Supplementary Information

Supplementary Figures

Supplementary Figure legends

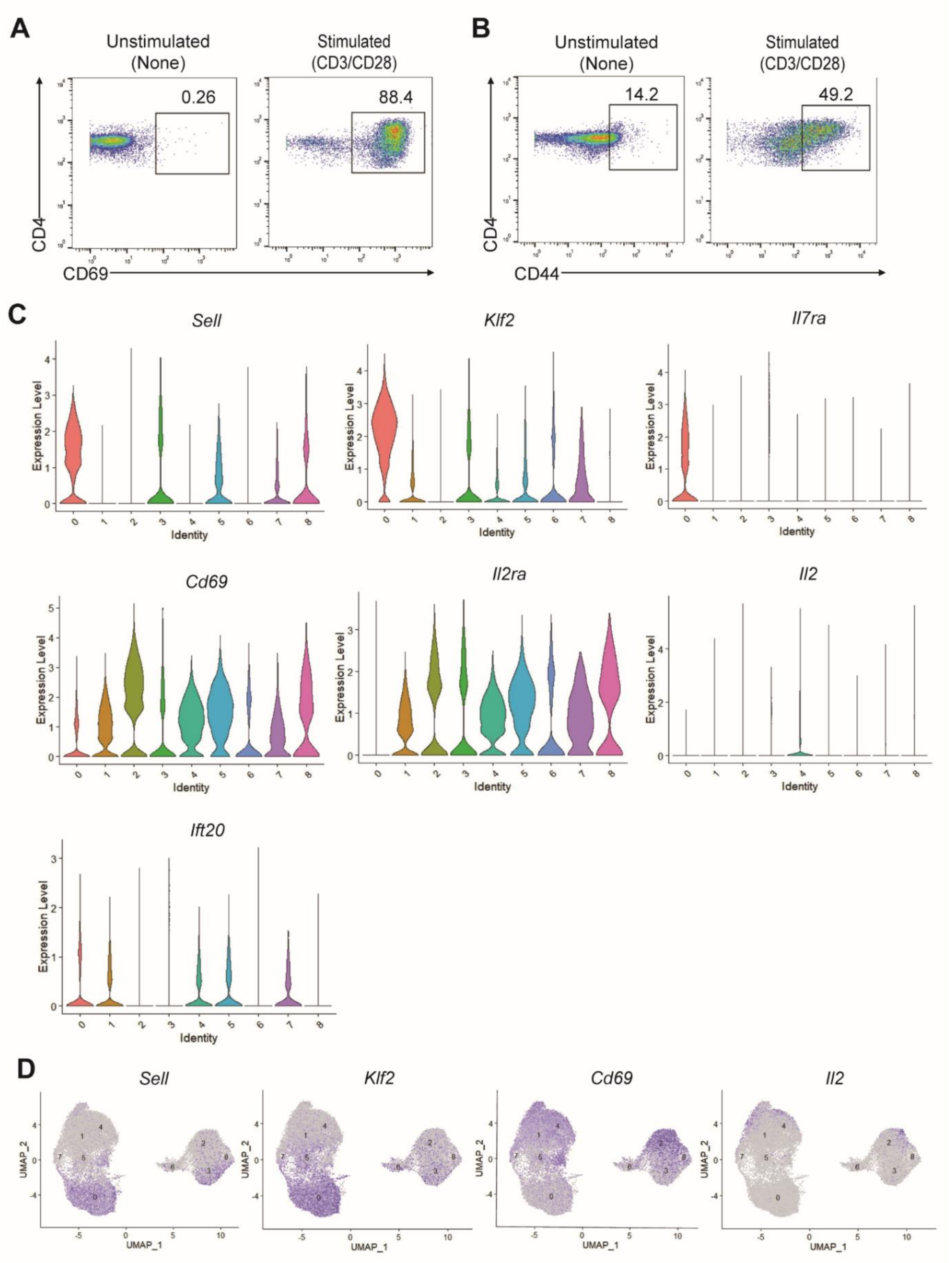

Supplementary Fig. 1. Single-cell RNA sequencing (scRNA-seq) and clustering strategy

A–B Activation of $CD4^+$ T cells in vitro. Naïve $CD4^+$ T cells enriched by magnetic beads were activated with 1-μg/mL anti-CD3 and 1-μg/mL anti-CD28 antibodies for 24 h. **A** Frequencies of $CD69^+$ $CD4^+$ T cells and **B** $CD44^+$ $CD4^+$ T cells were measured by flow cytometry. **C–D**

Analysis of scRNA-seq data from unstimulated and in vitro-activated CD4$^+$ T cells. **C** Violin plots *of Sell*, *Klf2*, *Il7ra*, *Cd69*, *Il2ra*, *Il2*, and *Ift20*. **D** Feature plots of *Sell*, *Klf2*, *Cd69*, and *Il2*. See also Fig. 1.

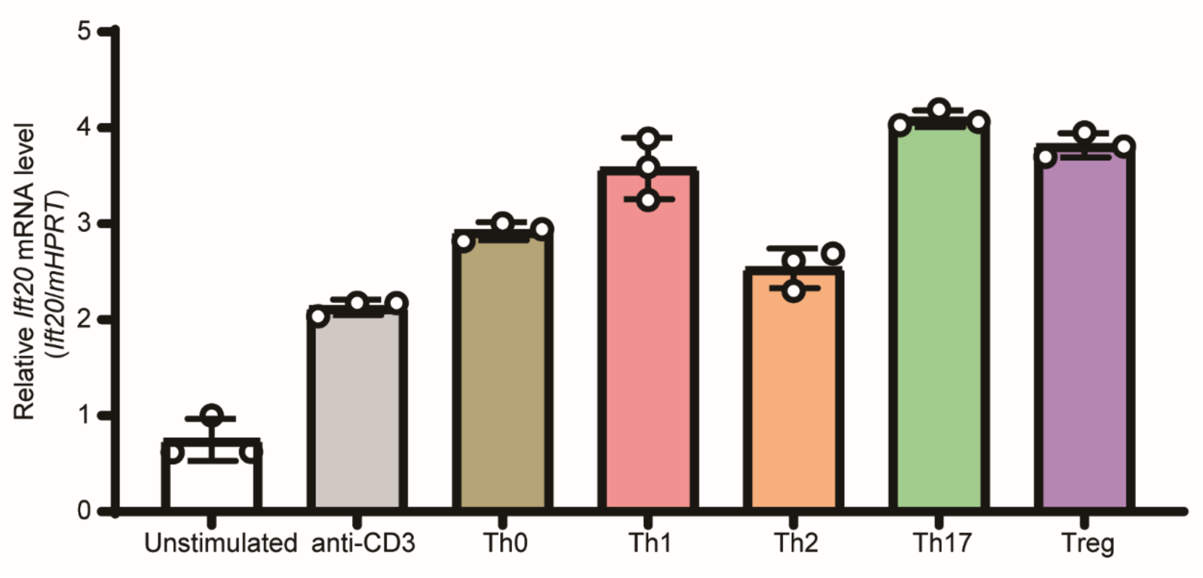

Supplementary Fig. 2. Alteration of *Ift20* mRNA expression by T cell differentiation

Naïve CD4$^+$ T cells were enriched by magnetic beads and activated with 1 μg/mL anti-CD3 antibodies, 1 μg/mL anti-CD28 antibodies, and skewing antibodies for 96 h. Skewing antibodies are as follows: anti-CD3 (1 μg/mL anti-CD3 antibodies); Th0 (1 μg/mL anti-CD3 antibodies and 1 μg/mL anti-CD28 antibodies); Th1 (1 μg/mL anti-CD3 antibodies, 1 μg/mL anti-CD28 antibodies, 5 μg/mL anti-IL-4 antibodies, 30 U/mL IL-2, and 10 ng/mL IL-12); Th2 (1 μg/mL anti-CD3 antibodies, 1 μg/mL anti-CD28 antibodies, 5 μg/mL anti-IFN-γ antibodies, 30 U/mL IL-2, and 10 ng/mL IL-4); Th17 (1 μg/mL anti-CD3 antibodies, 1 μg/mL anti-CD28 antibodies, 1 μg/mL anti-IFN-γ antibodies, 1 μg/mL anti-IL-4 antibodies, 20 ng/mL IL-6, and 6 ng/mL TGF-β1); Treg (1 μg/mL anti-CD3 antibodies, 1 μg/mL anti-CD28 antibodies, 5 μg/mL anti-IFN-γ antibodies, 5 μg/mL anti-IL-4 antibodies, 30 U/mL IL-2, and 30 ng/mL TGF-β1). *Ift20* mRNA was measured using quantitative PCR.

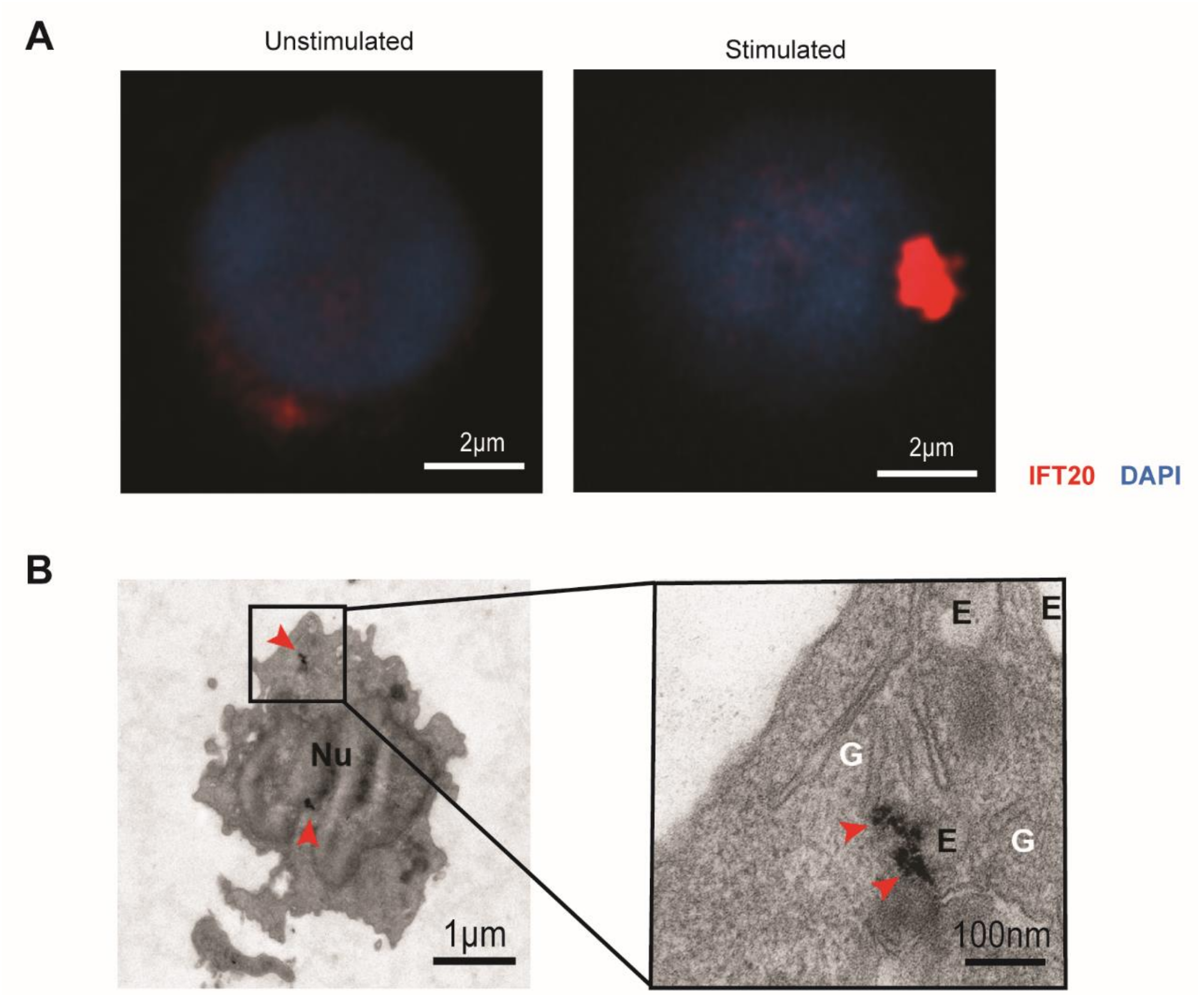

Supplementary Fig. 3. IFT20 localizes to the Golgi apparatus and the endosomes in immune cells

A Representative confocal microscopic image from unstimulated and stimulated $CD4^+$ T cells. Naïve $CD4^+$ T cells enriched by magnetic beads were activated with 1-μg/mL anti-CD3 and 1-μg/mL anti-CD28 antibodies for 8 h. Scale bar, 2 μm. **B** IFT20 immunogold labeling and transmission electron microscopy (TEM) analysis of immune cells in the mouse spleen: Red arrow, IFT20; Nu, nucleus; G, golgi apparatus; E, endosome. Scale bars, 1 μm and 100 nm, as indicated.

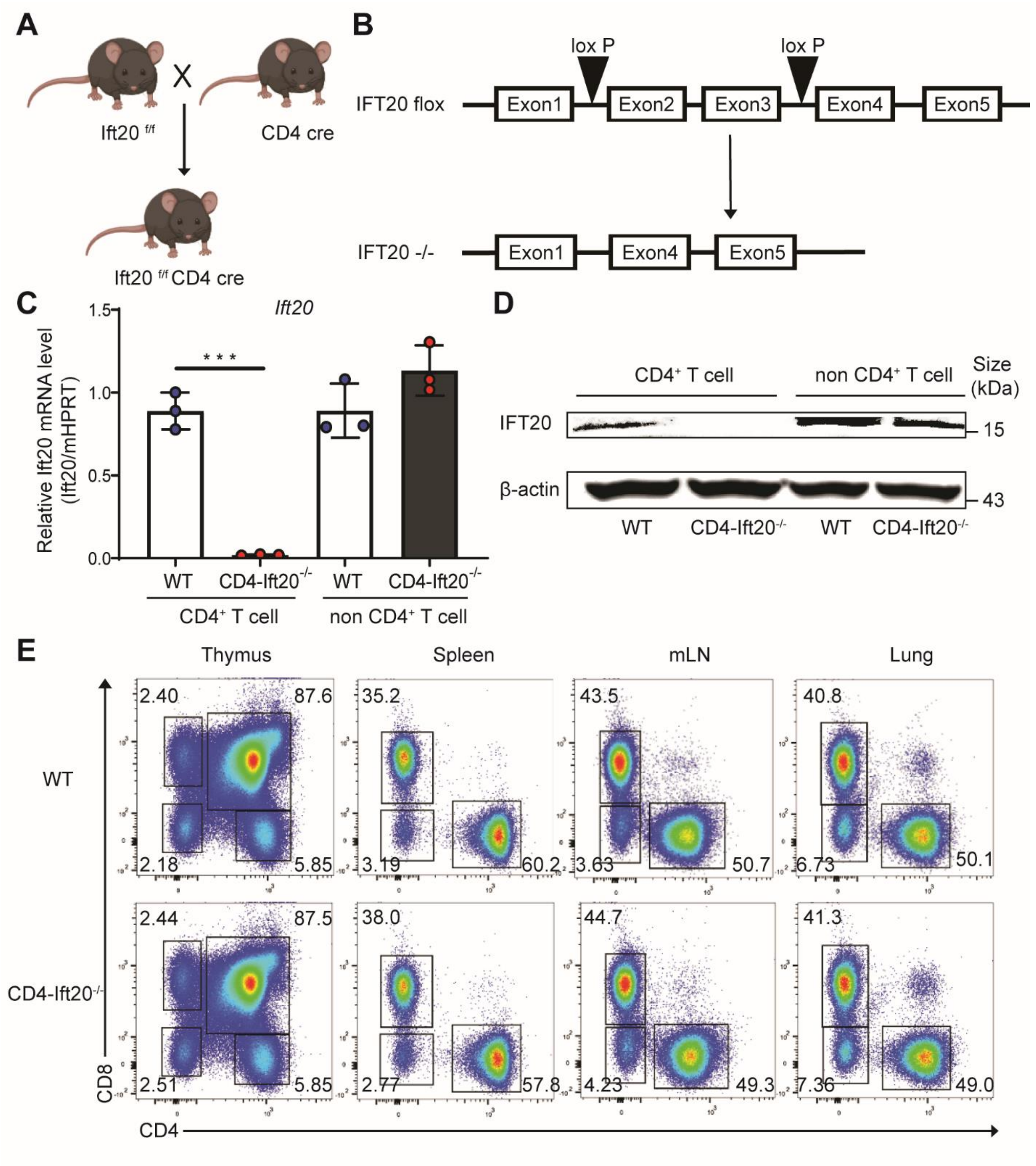

Supplementary Fig. 4. Generation of CD4^{+} T cell-specific IFT20-knockout (KO) mice

A An overview of breeding strategy for generating conditional IFT20-KO mice. **B** Structure of the Ift20 floxed and Ift20-/- alleles. **C** Levels of *Ift20* mRNA were measured by qRT-PCR in non-CD4^{+} T cells and CD4^{+} T cells enriched using magnetic beads from WT and IFT20-KO mice (n=3). **D** Levels of IFT20 protein were measured by western blot in non-CD4^{+} T cells and CD4^{+} T cells enriched using magnetic beads from WT and IFT20-KO mice. **E** The frequencies of CD4^{+} T cells, CD8^{+} T cells, double-positive cell (CD4^{+}CD8^{+} T cells), and double-negative cells (CD4^{-}CD8^{-} T cells) were measured in WT and IFT20-KO mice by flow cytometry. In **C-D**, the representative data were independently performed more than three times. CD4^{+} T cells was obtained by negative selection through the CD4^{+} T cell enrichment kit, and non-CD4^{+} T

cells refer to residual cells except sorted $CD4^+$ T cells. Data are shown as the mean ± SEM. Significance was calculated by the Student's t-test; *$P<0.05$, **$P<0.01$, ***$P<0.001$. See also Fig. 2.

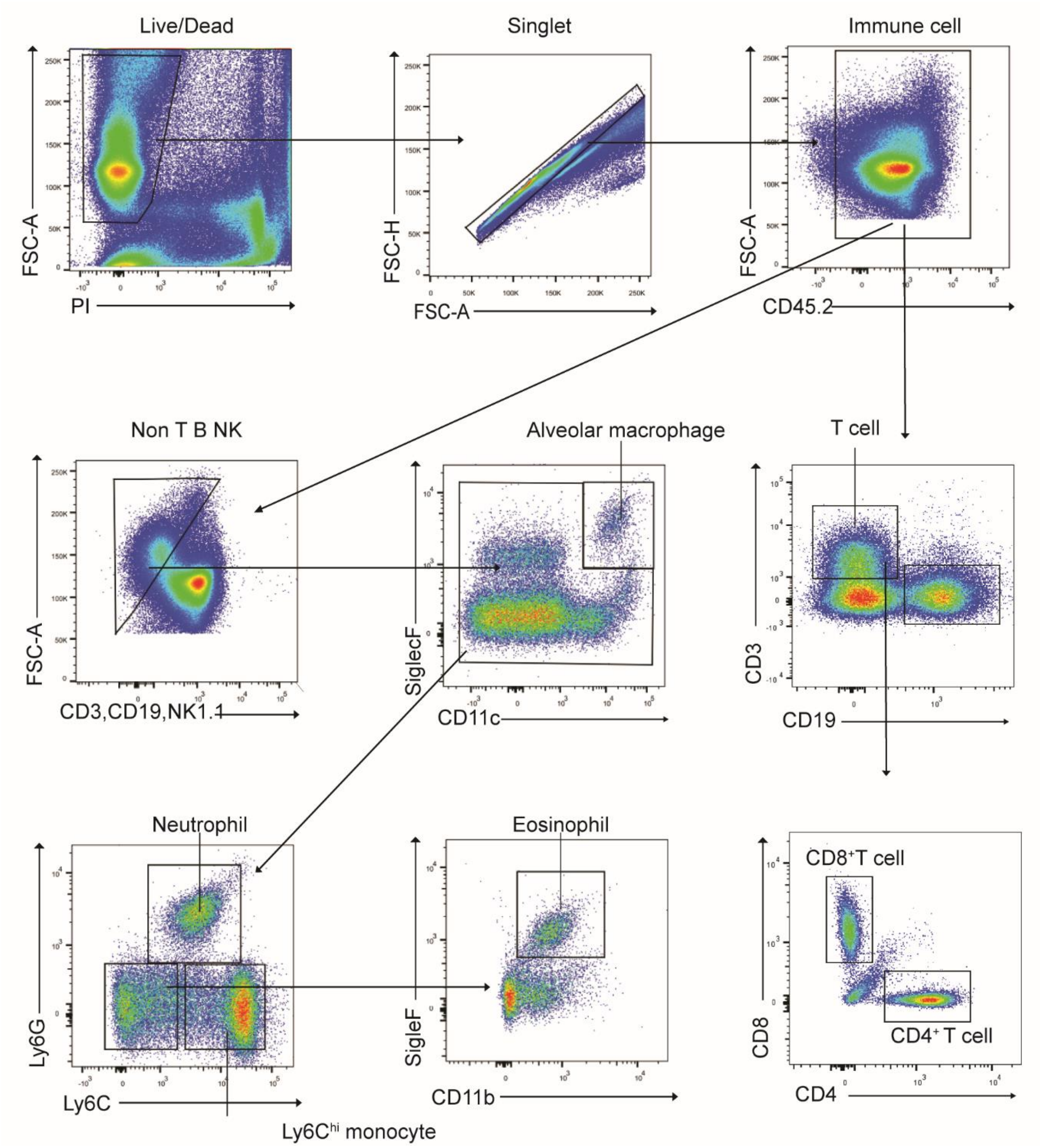

Supplementary Fig. 5. Gating strategy for the in vivo mouse model

CD4$^+$ T cell: CD45.2$^+$CD3$^+$CD4$^+$; CD8$^+$ T cell: CD45.2$^+$CD3$^+$CD8$^+$; Alveolar macrophage: CD45.2$^+$CD3$^-$CD19$^-$NK1.1$^-$SiglecF$^+$CD11c$^+$; Neutrophil: CD45.2$^+$CD3$^-$CD19$^-$NK1.1$^-$SiglecF$^-$Ly6G$^+$; Ly6C^{hi} monocyte: CD45.2$^+$CD3$^-$CD19$^-$NK1.1$^-$SiglecF$^-$Ly6G$^-$Ly6C$^+$; Eosinophil: CD45.2$^+$CD3$^-$CD19$^-$NK1.1$^-$Ly6G$^-$Ly6C$^-$SiglecF$^+$CD11b$^+$. See also Fig. 2 and Supplementary Fig. 6.

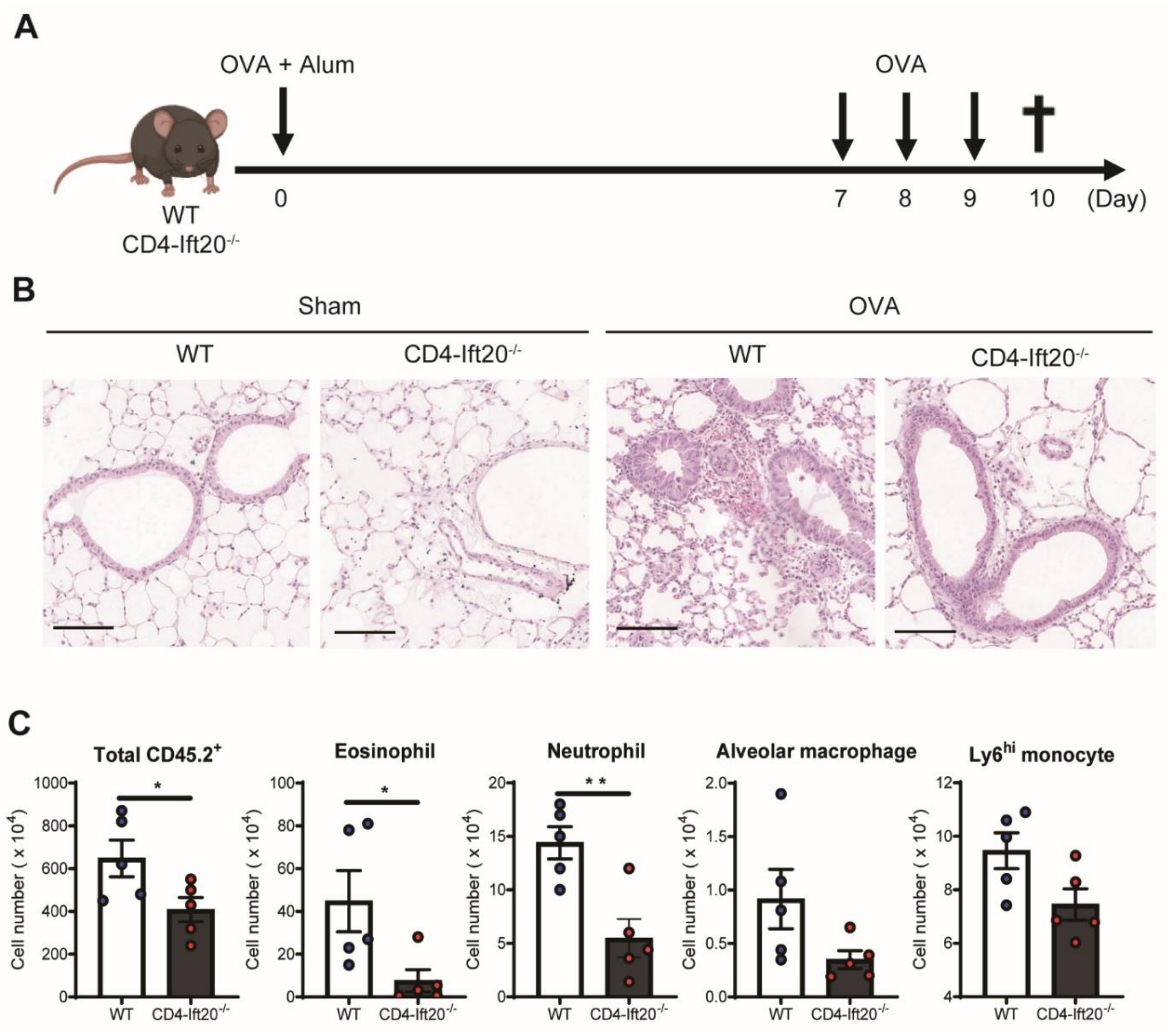

Supplementary Fig. 6. IFT20 deficiency alleviates ovalbumin (OVA)-induced airway inflammation

A An overview of the OVA-induced airway inflammation model. Mice were sensitized to 100 μg OVA and 4 mg alum via intraperitoneal injection on day 0. Animals were then intranasally challenged with 50 μg OVA on days 7–9 after the first intranasal injection, and lung tissue was harvested on day 10. **B** Representative hematoxylin and eosin (H&E) staining images of lung tissue from OVA-treated and sham control-treated WT and IFT20- KO mice. Scale bar, 100 μm. **C** Total numbers of CD45.2^{+} immune cells (n=5), eosinophils (n=5), neutrophils (n=5), alveolar macrophages (n=5), and Ly6hi monocytes (n=5) in the lungs of OVA-treated WT and IFT20-KO mice. In **B-C**, the data represent more than three independent experiments. Data are shown as the mean ± SEM. Significance was calculated using an unpaired Student's t-test. *P<0.05, **P<0.01, ***P<0.001.

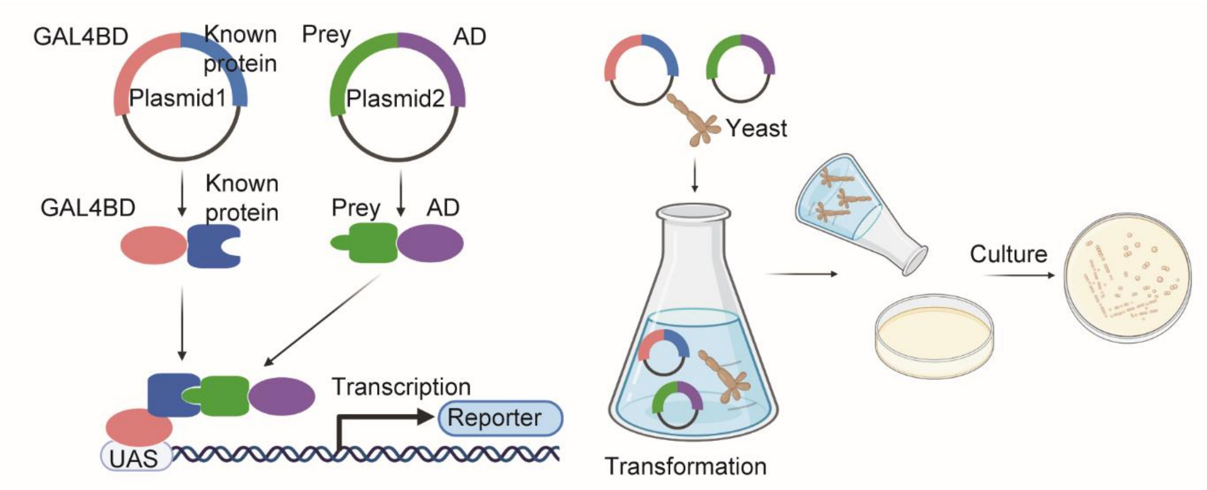

Supplementary Fig. 7. Schematic overview of the yeast two-hybrid (Y2H) system used to examine the interaction between TSG101 and IFT20

Known protein bait, Y2H protein screening prey, and proteins from the mouse spleen cDNA activation domain (AD) library bind to each other, leading to transcription and survival of cells on selective media. Yeast transformants containing the known protein bait plasmids, including IFT20 or TSG101, and proteins from the spleen AD library were spread on selective media. Yeast colonies growing on selective media were chosen based on reporter gene activity. UAS, upstream activating sequence; BD, binding domain; AD, activation domain.

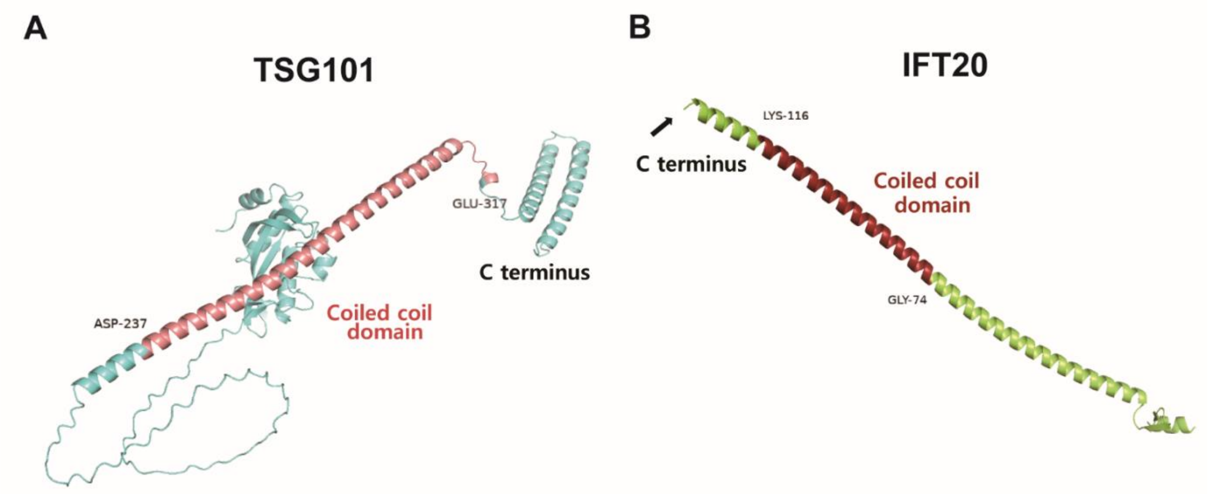

Supplementary Fig. 8. Protein structure analysis of IFT20 and TSG101

A The three-dimensional (3D) structure of TSG101. The coiled-coil domain consisting of aspartic acid (ASP) 237 to glutamate (GLU) 317 is presented in pink. **B** The 3D structure of IFT20. The coiled-coil domain consisting of glycine (GLY) 74 to lysine (LYS) 116 is presented in red. In **A-B**, PyMOL (Schrödinger, Mannheim, Germany) was used to generate the 3D structure of each protein.

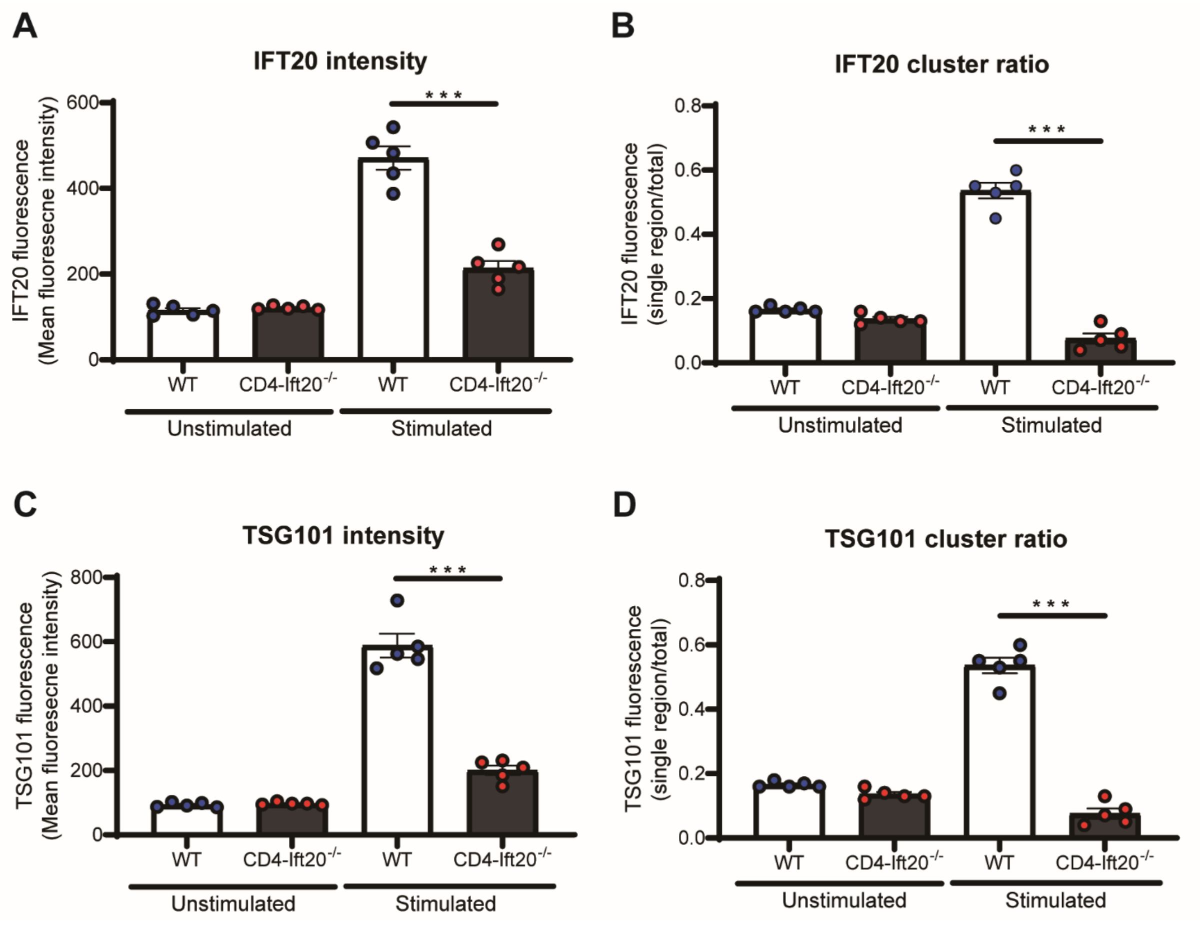

Supplementary Fig. 9. IFT20 acts as a critical regulator of central supramolecular activation cluster formation

A–D Naïve CD4$^+$ T cells from WT and IFT20-KO mice were enriched by magnetic beads and activated with 1-µg/mL anti-CD3 and 1-µg/mL anti-CD28 antibodies for 8 h (n=5). IFT20 intensities (**A**) and cluster ratios (**B**) and TSG101 intensities (**C**) and cluster ratios (**D**) were calculated using ZEN 3.2 software (Carl Zeiss, Oberkochen, Germany). See also Fig. 4.

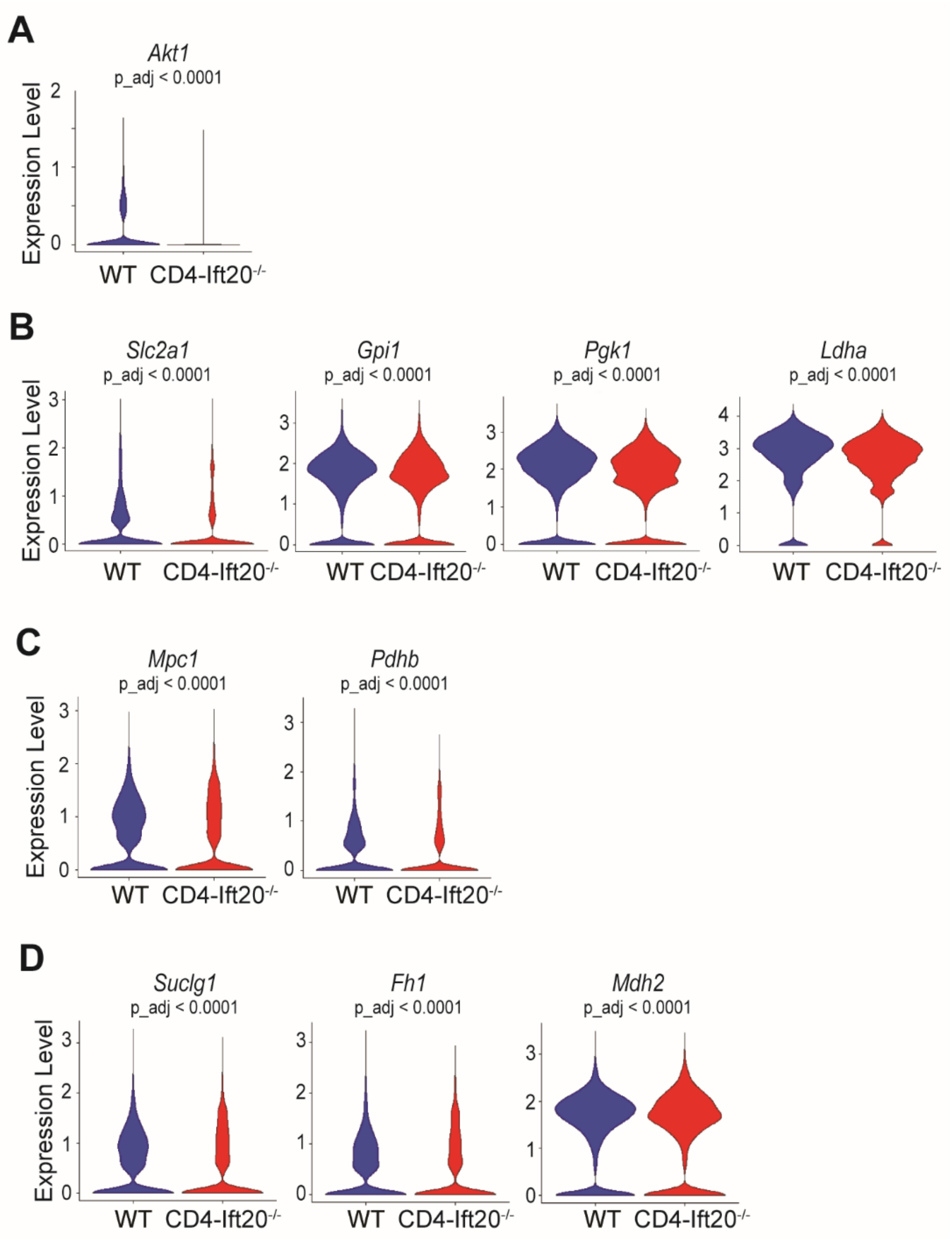

Supplementary Fig. 10. Differentially expressed genes in single-cell RNA sequencing

A-B Naïve CD4^{+} T cells from WT and IFT20-KO mice were activated with anti-CD3 and anti-CD28 for 24 h and analyzed by scRNA-seq. **A** Violin plots for the Akt-mTOR pathway related gene showing significant differential expression in the volcano plot in Fig. 5B. **B** Violin plots for the glycolysis related gene showing significant differential expression in the volcano plot

in Fig. 5B. **C** Violin plots for the pyruvate metabolism related gene showing significant differential expression in the volcano plot in Fig. 5B **D** Violin plots for the TCA cycle related gene showing significant differential expression in the volcano plot in Fig. 5B. See also Fig. 5.

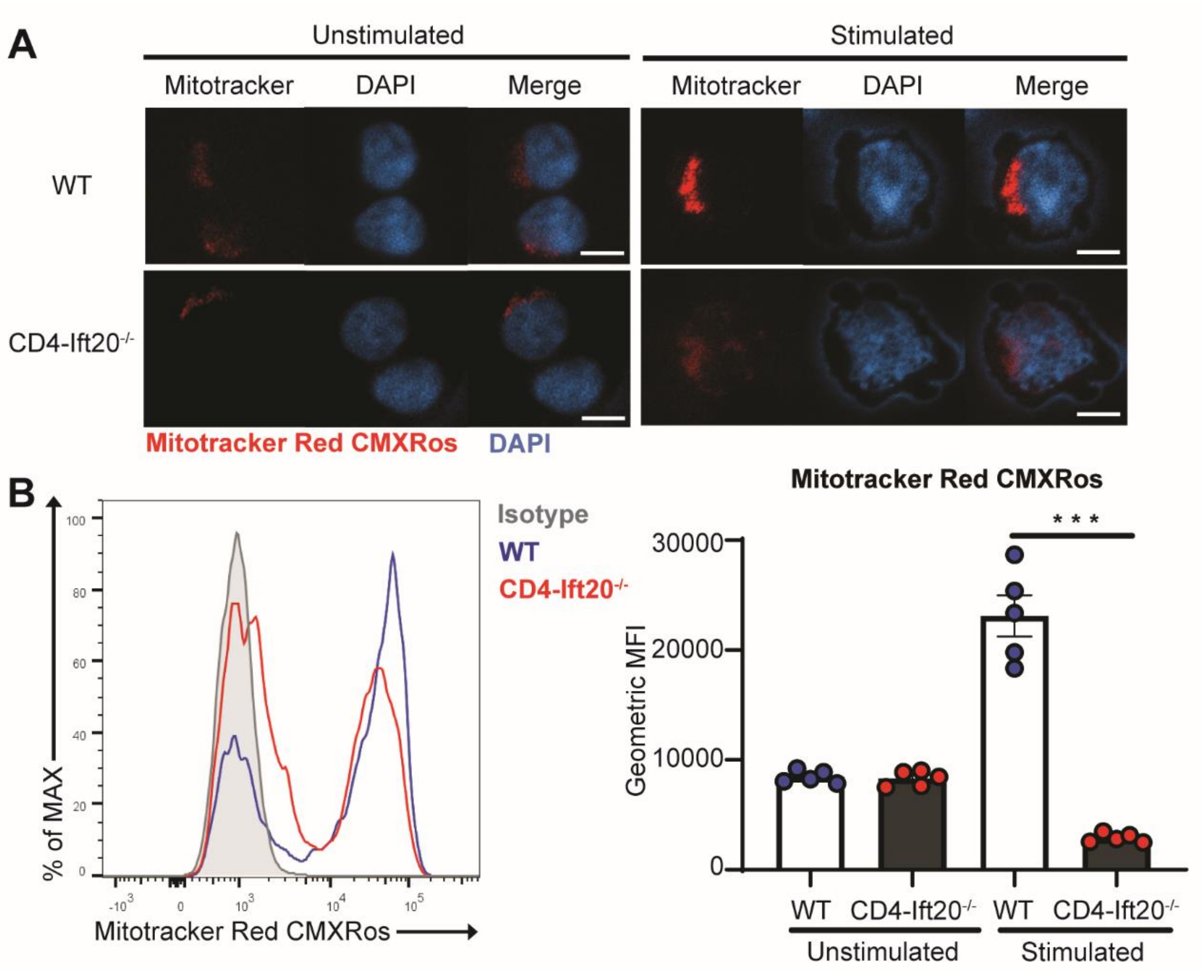

Supplementary Fig. 11. IFT20 regulates mitochondrial function in CD4+ T cells

Naïve CD4+ T cells from IFT20- KO and WT mice were enriched by magnetic beads and stimulated with 1-µg/mL anti-CD3 and 1-µg/mL anti-CD28 antibodies for 48 h. **A** Representative confocal microscopic images of unstimulated and stimulated CD4+ T cells from WT and IFT20-KO mice. Cells were stained with MitoTracker™ Red CMXRos (red), and 3D images were obtained using ZEN 3.2 software. Scale bar, 5 µm. **B** The mitochondrial membrane potential in stimulated CD4+ T cells from WT and IFT20-KO mice was assessed by flow cytometry. The representative mean fluorescence intensity (MFI) and bar graphs indicating the MFI levels are shown. Data are expressed as the mean ± SEM. Significance was calculated using an unpaired Student's t-test. ** P<0.01, *** P<0.001.

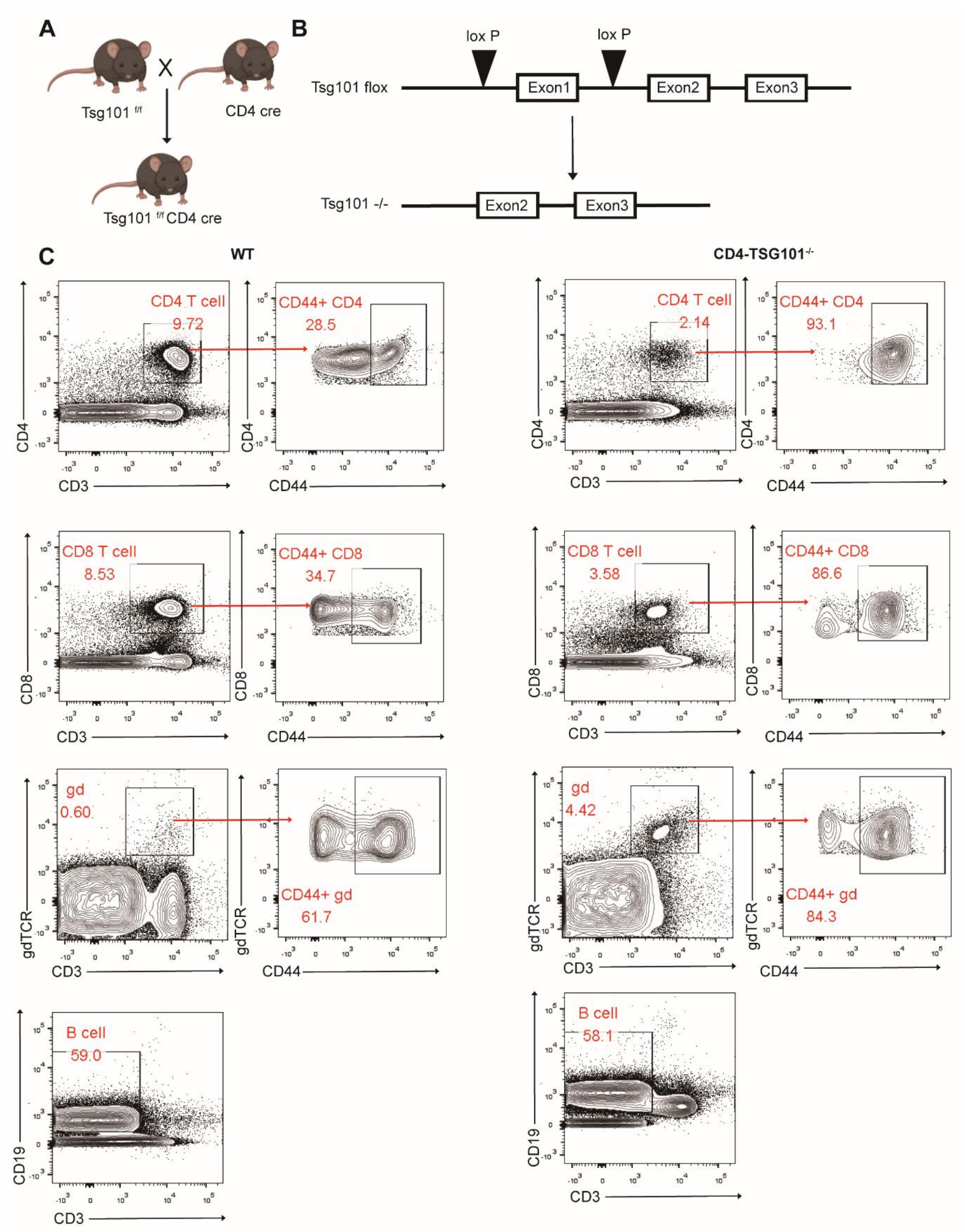

Supplementary Fig. 12. Generation of CD4$^+$ T cell-specific TSG101-KO mice

A An overview of the breeding strategy used to generate conditional TSG101-KO mice. **B** Structure of the *Tsg101* floxed and *Tsg101*$^{-/-}$ alleles. **C** The Frequencies of CD44 expressing cells in CD4$^+$, CD8$^+$ and γδ T cell compartment were measured in the spleens of WT and TSG101-KO mice by flow cytometry.

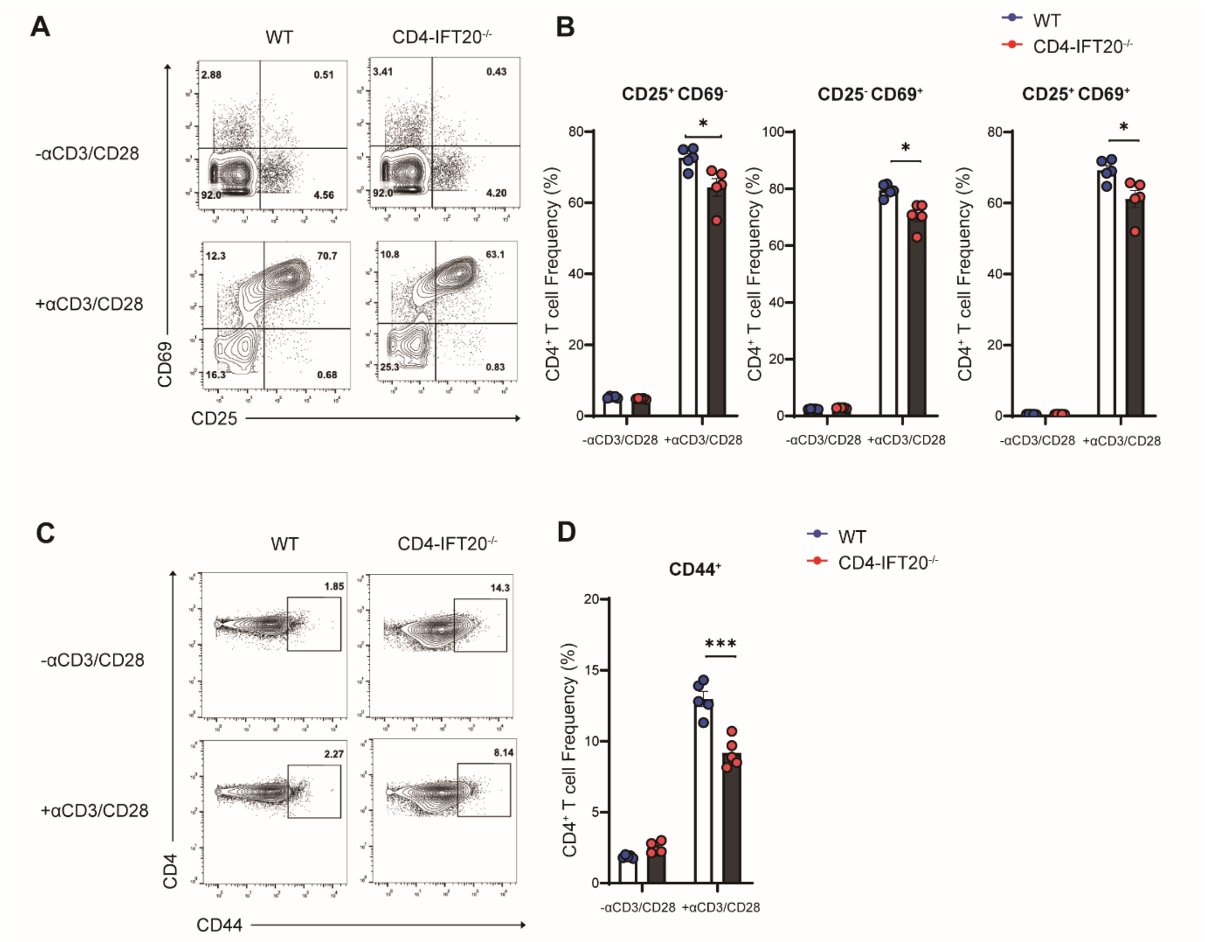

Supplementary Fig. 13. IFT20 deficiency reduced CD4+ T cell activation

A–D Naïve CD4+ T cells from the spleens of IFT20-KO and WT mice were enriched by magnetic beads and stimulated with 1-μg/mL anti-CD3 and 1-μg/mL anti-CD28 antibodies for 8 h. **A** Representative flow cytometry plots showing expression of CD25 and CD69 in unstimulated and stimulated CD4+ T cells from WT and IFT20-KO mice. **B** The frequencies of CD25+CD69- T cells, CD25-CD69+ T cells, and CD25+CD69+ T cells were measured by flow cytometry (n=5). **C** Representative flow cytometry plots showing expression of CD44 in unstimulated and stimulated CD4+ T cells from WT and IFT20-KO mice. **D** The frequencies of CD44+ T cells in CD4+ T cells were measured by flow cytometry (n=8). In **A–D,** data represent more than three independent experiments. CD4+ T cells were obtained by negative selection using a naïve CD4+ T cell enrichment kit. Data are shown as the mean ± SEM. Significance was calculated using Student's t-test; * P<0.05, ***P<0.001.
